# Association between COVID-19 convalescent plasma antibody levels and COVID-19 outcomes stratified by clinical status at presentation

**DOI:** 10.1186/s12879-024-09529-0

**Published:** 2024-06-26

**Authors:** Hyung Park, Chang Yu, Liise-anne Pirofski, Hyunah Yoon, Danni Wu, Yi Li, Thaddeus Tarpey, Eva Petkova, Elliott M. Antman, Andrea B. Troxel, the COMPILE Consortium

**Affiliations:** 1 Department of Population Health, NYU Grossman School of Medicine, New York, NY USA; 2 Department of Medicine, Division of Infectious Diseases, Albert Einstein College of Medicine and Montefiore Medical Center, Bronx, NY USA; 3 Department of Child and Adolescent Psychiatry, NYU Grossman School of Medicine, New York, NY USA; 4 Brigham and Women’s Hospital, Harvard Medical School, Boston, MA USA

**Keywords:** COVID-19, Convalescent plasma, SARS-CoV-2 IgG level, Dose-response analysis

## Abstract

**Background:**

There is a need to understand the relationship between COVID-19 Convalescent Plasma (CCP) anti-SARS-CoV-2 IgG levels and clinical outcomes to optimize CCP use. This study aims to evaluate the relationship between recipient baseline clinical status, clinical outcomes, and CCP antibody levels.

**Methods:**

The study analyzed data from the COMPILE study, a meta-analysis of pooled individual patient data from 8 randomized clinical trials (RCTs) assessing the efficacy of CCP vs. control, in adults hospitalized for COVID-19 who were not receiving mechanical ventilation at randomization. SARS-CoV-2 IgG levels, referred to as ‘dose’ of CCP treatment, were retrospectively measured in donor sera or the administered CCP, semi-quantitatively using the VITROS Anti-SARS-CoV-2 IgG chemiluminescent immunoassay (Ortho-Clinical Diagnostics) with a signal-to-cutoff ratio (S/Co). The association between CCP dose and outcomes was investigated, treating dose as either continuous or categorized (higher vs. lower vs. control), stratified by recipient oxygen supplementation status at presentation.

**Results:**

A total of 1714 participants were included in the study, 1138 control- and 576 CCP-treated patients for whom donor CCP anti-SARS-CoV2 antibody levels were available from the COMPILE study. For participants not receiving oxygen supplementation at baseline, higher-dose CCP (/control) was associated with a reduced risk of ventilation or death at day 14 (OR = 0.19, 95% CrI: [0.02, 1.70], posterior probability Pr(OR < 1) = 0.93) and day 28 mortality (OR = 0.27 [0.02, 2.53], Pr(OR < 1) = 0.87), compared to lower-dose CCP (/control) (ventilation or death at day 14 OR = 0.79 [0.07, 6.87], Pr(OR < 1) = 0.58; and day 28 mortality OR = 1.11 [0.10, 10.49], Pr(OR < 1) = 0.46), exhibiting a consistently positive CCP dose effect on clinical outcomes. For participants receiving oxygen at baseline, the dose-outcome relationship was less clear, although a potential benefit for day 28 mortality was observed with higher-dose CCP (/control) (OR = 0.66 [0.36, 1.13], Pr(OR < 1) = 0.93) compared to lower-dose CCP (/control) (OR = 1.14 [0.73, 1.78], Pr(OR < 1) = 0.28).

**Conclusion:**

Higher-dose CCP is associated with its effectiveness in patients not initially receiving oxygen supplementation, however, further research is needed to understand the interplay between CCP anti-SARS-CoV-2 IgG levels and clinical outcome in COVID-19 patients initially receiving oxygen supplementation.

**Supplementary Information:**

The online version contains supplementary material available at 10.1186/s12879-024-09529-0.

## Introduction

In April 2020, amid the onset of the COVID-19 pandemic in the United States, a single-arm Expanded Access Program sponsored by the Mayo Clinic provided access to COVID-19 convalescent plasma (CCP) for hospitalized patients with COVID-19. The outcomes of patients treated with CCP suggested a dose-response relationship, whereby administration of CCP deemed ‘high titer’ compared to CCP deemed ‘low titer’ was associated with reduced mortality when given within 72 h of hospital admission to non-intubated patients [1]. 

Several randomized controlled trials (RCTs) assessed the efficacy of CCP (we refer to Franchini et al. [2] and Kimber et al. [3] for a review of the RCTs). Most trials involving hospitalized patients with severe COVID-19 showed no overall clinical benefit (CONTAIN Covid-19^4^; RECOVERY [5]; PlasmAr [6]; PLACID [7]). However, some trials that used high-titer CCP in hospitalized patients reported a mortality benefit [1, 8–10], and others found clinical benefit for outpatients [11–13]. Focosi et al. [14] suggested a greater clinical benefit when CCP neutralizing titer exceeded 1:160 and time to randomization from symptoms onset was under 9 days, highlighting that best results are obtained when high-titer CCP is administered early in COVID-19. In a study of severely ill patients, Rojas et al. [15] noted a shorter hospital stay with CCP treatment, but no significant CCP impact on ICU demand, mechanical ventilation, or mortality. However, Misset et al. [16] found a mortality benefit in mechanically ventilated patients who received high-titer CCP with a neutralizing titer of at least 1:160, when administered within 48 h of ventilation initiation.

Notably, most studies that did not find a CCP benefit were limited by a lack of consideration of biological plausibility in trial design, because they enrolled hospitalized patients, particularly those receiving oxygen supplementation at enrollment, and/or used CCP that was not high titer. However, data from the pandemic associate the benefit of CCP with use of high titer CCP early in the disease [14, 17].

Early in the COVID-19 pandemic, the Continuous Monitoring of Pooled International Trials of Convalescent Plasma for COVID-19 Hospitalized Patients (COMPILE) Consortium [18, 19] was established. The consortium was comprised of RCTs of CCP for the treatment of hospitalized patients with COVID-19 who were not receiving mechanical ventilation at the time of randomization. Participants were enrolled from early 2020 to March 2021 and observed until day 28 ± 2 after treatment initiation. Their clinical status was assessed using the 11-point WHO clinical status scale [20] (Supplementary Figure S1) both at baseline and at the primary endpoint assessment time of 14 days. The COMPILE database was locked in April 2021. Ultimately, 8 RCTs from 6 countries and 4 continents provided data on 2341 participants. The main results of the study were published in Troxel et al. [18]. Although the aggregate analysis of COMPILE did not reveal a significant overall benefit of CCP, it was likely beneficial in participants who did not require oxygen supplementation at enrollment (WHO Clinical Progression Scale score of 4) (Supplementary Table S1). In addition, Park et al. [21]. derived a treatment-benefit-index (TBI) using patient age, comorbidities, and oxygen supplementation status to identify patients who might benefit from CCP treatment. In Park et al. [21], the efficacy of CCP was analyzed using only a binary CCP treatment indicator without knowledge of CCP SARS-CoV-2 IgG level (referred to as the ‘dose’ of CCP treatment) because data on CCP dose was not available at the time. In the present paper, we analyze the CCP dose-outcome association, stratified by WHO score-defined oxygen supplementation status at baseline, hypothesizing that the association is modified by the severity of COVID-19, as indicated by oxygen requirements, at presentation.

## Methods

CCP SARS-CoV-2 spike protein-binding antibody levels were measured retrospectively using donor sera obtained at the time of donation or the administered CCP. Semi-quantitative measurements were obtained with the Ortho Clinical Diagnostics VITROS® XT7600 Integrated System Anti-SARS-CoV-2 IgG assay (OrthoV), by each participating COMPILE RCT following the manufacturer’s protocol. See Supplementary Materials S4.3 for details. The CCP dose analysis follows the outline in Sect. 9.3.3 of the COMPILE study’s Statistical Analysis Plan (included as Supplementary Materials) [18]. The association between CCP dose and clinical outcomes was assessed, treating dose as either a continuous or categorical variable (higher vs. lower, categorized at the sample mean OrthoV S/Co value, or control). The association was stratified by recipient oxygen supplementation status at baseline (the time of randomization) to analyze the differential association between CCP dose and outcomes by disease severity at presentation.

Both continuous and categorized dose-response regression analyses were adjusted for the participating RCT, age, sex, patient enrollment quarter, concomitant medications, and baseline patient characteristics reported in Table 1. CCP-treated patients with missing OrthoV antibody measurements were excluded from the analysis. Missing baseline covariates were imputed 100 times and the results were subsequently combined (Supplementary Section S1). We refer to Supplementary Section S2.3 for a sensitivity analysis addressing potential selection bias due to excluding patients with missing antibody measures. Day 14 and day 28 outcomes on the 11-point WHO scale were modeled by cumulative logit proportional odds regression, whereas dichotomized outcomes (i.e., incidence of ventilation or death at day 14 or day 28, and mortality at day 28) were modeled using logistic regression. The continuous dose-response analysis, treating OrthoV IgG levels as a continuous variable, was conducted among the CCP-treated participants, modeling the impact of different doses (i.e., different OrthoV levels) by restricted cubic splines to accommodate possibly nonlinear dose impacts (details in Supplementary Section S2.1). The categorized dose-response association analysis, comparing higher vs. lower doses, included COMPILE control arm participants as a comparison group. Bayesian multivariable regression utilized the dose group as the main regressor with three levels (higher vs. lower dose vs. control), adjusting for the baseline variables in Table 1, employing weakly informative priors for conservative parameter estimation to reduce type I error rates and mitigate the need for post-hoc corrections for multiple comparisons (details in Supplementary Section S2.1). Additionally, interactions between dose and TBI, as well as with patient baseline covariates, were explored as part of the analysis (details in Supplementary Sections S2.2 and S3.2).

## Results

**Fig. 1:**
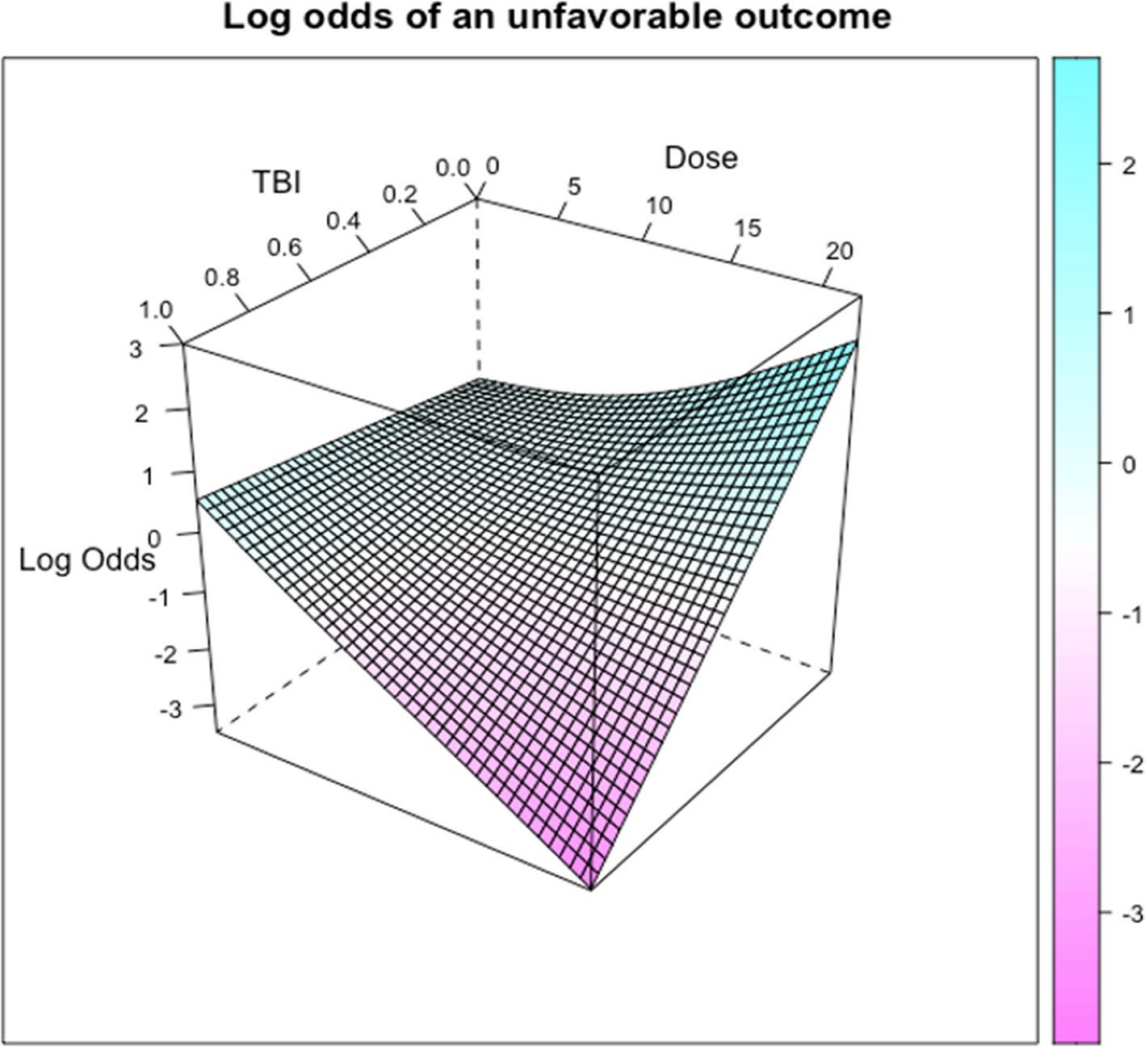
Log odds of ventilation or death on day 14 post-treatment in the TBI-dose domain (with lower odds clinically desirable)

The analysis included 1714 COVID-19 hospitalized patients not receiving ventilatory support at the time of randomization, comprising 1138 control and 576 CCP-treated participants with available CCP OrthoV IgG measurements (Table 1). Supplementary Table S2 shows a summary of CCP antibody levels. Figure 1 below displays the log odds of ventilation or death (i.e., of an unfavorable outcome) on day 14 post-treatment as a surface over the TBI (0–1 range, developed in Park et al. [21]) value on the x-axis, and the CCP SARS-CoV-2 IgG ‘dose’ (measured in OrthoV units) on the y-axis, adjusted for the participating RCT, participant enrollment quarter and concomitant medications (logistic regression with restricted cubic splines allowed for the examination of a nonlinear interaction between TBI and dose).

**Table 1:** Summary of participant baseline (at enrollment) variables, including baseline characteristics, pre-existing health conditions, and concomitant medications, stratified by CCP dose groups (control, lower, higher, and missing OrthoV measurements). The last column reports p-values for differences between lower dose (< 8 OrthoV S/Co) and higher dose (≥ 8 OrthoV S/Co) groups, determined using chi-square association tests for each baseline variable

Baseline variable	Control (*n* = 1138)	Lower dose (< 8 OrthoV S/Co) (*n* = 304)	Higher dose (≥ 8 OrthoV S/Co) (*n* = 272)	Missing antibody measures (*n* = 655)	*P*-value (Lower vs. Higher dose)
Individual RCT (%)					**< 0.001**
- NYC	473 (41.6%)	104 (34.2%)	37 (13.6%)	327 (49.9%)	
- UPenn	39 (3.4%)	28 (9.2%)	11 (4.0%)	2 (0.3%)	
- Spain	171 (15.0%)	85 (28.0%)	90 (33.1%)	4 (0.6%)	
- UCSF	18 (1.6%)	0 (0.0%)	16 (5.9%)	0 (0.0%)	
- Belgium	163 (14.3%)	70 (23.0%)	81 (29.8%)	163 (24.9%)	
- Brazil	15 (1.3%)	10 (3.3%)	7 (2.6%)	2 (0.3%)	
- Netherlands	35 (3.1%)	7 (2.3%)	30 (11.0%)	0 (0.0%)	
- India	224 (19.7%)	0 (0.0%)	0 (0.0%)	157 (24.0%)	
Age (%)					0.489
- Age ≤ 50	287 (25.2%)	69 (22.7%)	72 (26.5%)	185 (28.2%)	
- 50 < Age ≤ 65	421 (37.0%)	97 (31.9%)	88 (32.4%)	250 (38.2%)	
- Age > 65	430 (37.8%)	138 (45.4%)	112 (41.2%)	220 (33.6%)	
Sex (%)					0.594
- Male	730 (64.1%)	188 (61.8%)	175 (64.3%)	431 (65.8%)	
- Female	408 (35.9%)	116 (38.2%)	97 (35.7%)	224 (34.2%)	
Baseline WHO score (%)					**0.006**
- 4 (hospitalized but no oxygen therapy)	235 (20.7%)	32 (10.5%)	45 (16.5%)	140 (21.4%)	
- 5 (oxygen by mask or nasal prong)	701 (61.6%)	212 (69.7%)	196 (72.1%)	392 (59.8%)	
- 6 (oxygen by high flow or non-invasive ventilation)	202 (17.8%)	60 (19.7%)	31 (11.4%)	123 (18.8%)	
Recipient blood group (%)					0.051
- O	518 (45.5%)	157 (51.6%)	113 (41.5%)	298 (45.5%)	
- A	374 (32.9%)	106 (34.9%)	109 (40.1%)	205 (31.3%)	
- B	195 (17.1%)	26 (8.6%)	36 (13.2%)	119 (18.2%)	
- AB	38 (3.3%)	13 (4.3%)	8 (2.9%)	33 (5.0%)	
- NA	13 (1.1%)	2 (0.7%)	6 (2.2%)	0 (0.0%)	
Systolic blood pressure > 127 mmHg (%)					0.833
- No	364 (32.0%)	88 (28.9%)	69 (25.4%)	254 (38.8%)	
- Yes	282 (24.8%)	96 (31.6%)	70 (25.7%)	229 (35.0%)	
- NA	492 (43.2%)	120 (39.5%)	133 (48.9%)	172 (26.3%)	
Weight > 90 kg (%)					0.584
- No	460 (40.4%)	142 (46.7%)	121 (44.5%)	276 (42.1%)	
- Yes	286 (25.1%)	91 (29.9%)	68 (25.0%)	204 (31.1%)	
- NA	392 (34.4%)	71 (23.4%)	83 (30.5%)	175 (26.7%)	
History of asthma (%)					1.000
- No	779 (68.5%)	257 (84.5%)	228 (83.8%)	430 (65.6%)	
- Yes	75 (6.6%)	19 (6.2%)	16 (5.9%)	64 (9.8%)	
- NA	284 (25.0%)	28 (9.2%)	28 (10.3%)	161 (24.6%)	
History of diabetes (all types) (%)					0.355
- No	770 (67.7%)	211 (69.4%)	178 (65.4%)	415 (63.4%)	
- Yes	368 (32.3%)	93 (30.6%)	94 (34.6%)	240 (36.6%)	
History of pulmonary disease (all types) (%)					0.983
- No	998 (87.7%)	259 (85.2%)	232 (85.3%)	591 (90.2%)	
- Yes	136 (12.0%)	45 (14.8%)	39 (14.3%)	60 (9.2%)	
- NA	4 (0.4%)	0 (0.0%)	1 (0.4%)	4 (0.6%)	
History of cardiovascular disease (all types) (%)					0.123
- No	660 (58.0%)	137 (45.1%)	141 (51.8%)	416 (63.5%)	
- Yes	474 (41.7%)	167 (54.9)	131 (48.2%)	236 (36.0%)	
- NA	4 (0.4%)	0 (0.0%)	0 (0.0%)	3 (0.5%)	
Days since symptom onset (%)					0.257
- 0–3	142 (12.5%)	46 (15.1%)	30 (11.0%)	72 (11.0%)	
- 4–6	394 (34.6%)	127 (41.8%)	107 (39.3%)	207 (31.6%)	
- 7–10	402 (35.3%)	95 (31.2%)	95 (34.9%)	241 (36.8%)	
- 11–14	136 (12.0%)	21 (6.9%)	19 (7.0%)	85 (13.0%)	
- > 14	58 (5.1%)	10 (3.3%)	17 (6.2%)	47 (7.2%)	
- NA	6 (0.5%)	5 (1.6%)	4 (1.5%)	3 (0.5%)	
Quarter of randomization (%)					**< 0.001**
- Apr-June 2020	344 (30.2%)	31 (10.2)	66 (24.3%)	200 (30.5%)	
- July-Sept 2020	215 (18.9%)	57 (18.8)	62 (22.8%)	123 (18.8%)	
- Oct-Dec 2020	405 (35.6%)	185 (60.9)	118 (43.4%)	201 (30.7%)	
- Jan-Mar 2021	174 (15.3%)	31 (10.2)	26 (9.6%)	131 (20.0%)	
Antiplatelet agents (%)					0.131
- No	539 (47.4%)	148 (48.7%)	131 (48.2%)	367 (56.0%)	
- Yes	145 (12.7%)	43 (14.1%)	24 (8.8%)	124 (18.9%)	
- NA	454 (39.9%)	113 (37.2%)	117 (43.0%)	164 (25.0%)	
Anticoagulant agents (%)					0.578
- No	372 (32.7%)	102 (33.6%)	83 (30.5%)	301 (46.0%)	
- Yes	456 (40.1%)	160 (52.6%)	147 (54.0%)	194 (29.6%)	
- NA	310 (27.2%)	42 (13.8%)	42 (15.4%)	160 (24.4%)	
Serostatus positive (%)					0.425
- No	267 (23.5%)	91 (29.9%)	85 (31.2%)	161 (24.6%)	
- Yes	354 (31.1%)	84 (27.6%)	64 (23.5%)	185 (28.2%)	
- NA	517 (45.4%)	129 (42.4%)	123 (45.2%)	309 (47.2%)	
Hydroxychloroquine (%)					**0.007**
- No	919 (80.8%)	292 (96.1%)	245 (90.1%)	531 (81.1%)	
- Yes	218 (19.2%)	12 (3.9%)	27 (9.9%)	124 (18.9%)	
- NA	1 (0.1%)	0 (0.0%)	0 (0.0%)	0 (0.0%)	
Antibacterial (%)					0.610
- No	534 (46.9%)	177 (58.2%)	165 (60.7%)	304 (46.4%)	
- Yes	603 (53.0%)	127 (41.8%)	107 (39.3%)	350 (53.4%)	
- NA	1 (0.1%)	0 (0.0%)	0 (0.0%)	1 (0.2%)	
Antiviral (not remdesivir)(%)					**0.014**
- No	980 (86.1%)	281 (92.4%)	214 (78.7%)	592 (90.4%)	
- Yes	123 (10.8%)	16 (5.3%)	28 (10.3%)	63 (9.6%)	
- NA	35 (3.1%)	7 (2.3%)	30 (11.0%)	0 (0.0%)	
Remdesivir (%)					0.115
- No	710 (62.4%)	182 (59.9%)	165 (60.7%)	448 (68.4%)	
- Yes	381 (33.5%)	115 (37.8%)	77 (28.3%)	198 (30.2%)	
- NA	47 (4.1%)	7 (2.3%)	30 (11.0%)	9 (1.4%)	
Anti-inflammatory (non-steroids) (%)					**0.003**
- No	914 (80.3%)	250 (82.2%)	248 (91.2%)	534 (81.5%)	
- Yes	203 (17.8%)	53 (17.4%)	24 (8.8%)	113 (17.3%)	
- NA	21 (1.8%)	1 (0.3%)	0 (0.0%)	8 (1.2%)	
Steroids (%)					**0.023**
- No	275 (24.2%)	73 (24.0%)	82 (30.1%)	154 (23.5%)	
- Yes	822 (72.2%)	224 (73.7%)	160 (58.8%)	496 (75.7%)	
- NA	41 (3.6%)	7 (2.3%)	30 (11.0%)	5 (0.8)	
Antithrombotic (%)					**0.008**
- No	235 (20.7%)	70 (23.0%)	83 (30.5%)	72 (11.0%)	
- Yes	861 (75.7%)	227 (74.7%)	159 (58.5%)	582 (88.9%)	
- NA	42 (3.7%)	7 (2.3%)	30 (11.0%)	1 (0.2%)	
TBI^a^ (%)					0.352
- Low (< 0.35)	674 (59.2%)	194 (63.8%)	166 (61.0%)	401 (61.2%)	
- High (≥ 0.35)	436 (38.3%)	108 (35.5%)	101 (37.1%)	238 (36.3%)	
- NA	28 (2.5%)	2 (0.7%)	5 (1.8%)	16 (2.4%)	

^a^Treatment-benefit-index (TBI) is a patient-specific score used to predict the potential therapeutic effectiveness of CCP treatment (vs. control), calculated based on pre-treatment patient characteristics, including baseline symptoms severity (oxygen support status defined from the WHO score), age, patient blood type, history of diabetes, cardiovascular and pulmonary disease (see Table S1 in Supplementary Materials). Higher TBI scores (e.g., those that belong to the “High CCP benefit” group; defined as TBI ≥ 0.35 in the table) indicate a predicted greater likelihood of therapeutically favorable CCP response compared to control. Except for TBI which is a data-driven composite variable, all baseline variables reported in this table were used as adjusting variables

These data in Fig. 1 indicate that with a high TBI value (close to 1, indicating a high predicted likelihood of benefiting from CCP), a higher CCP dose leads to a more favorable outcome, suggesting a dose-response relationship for patients with a high TBI value.

**Fig. 2:**
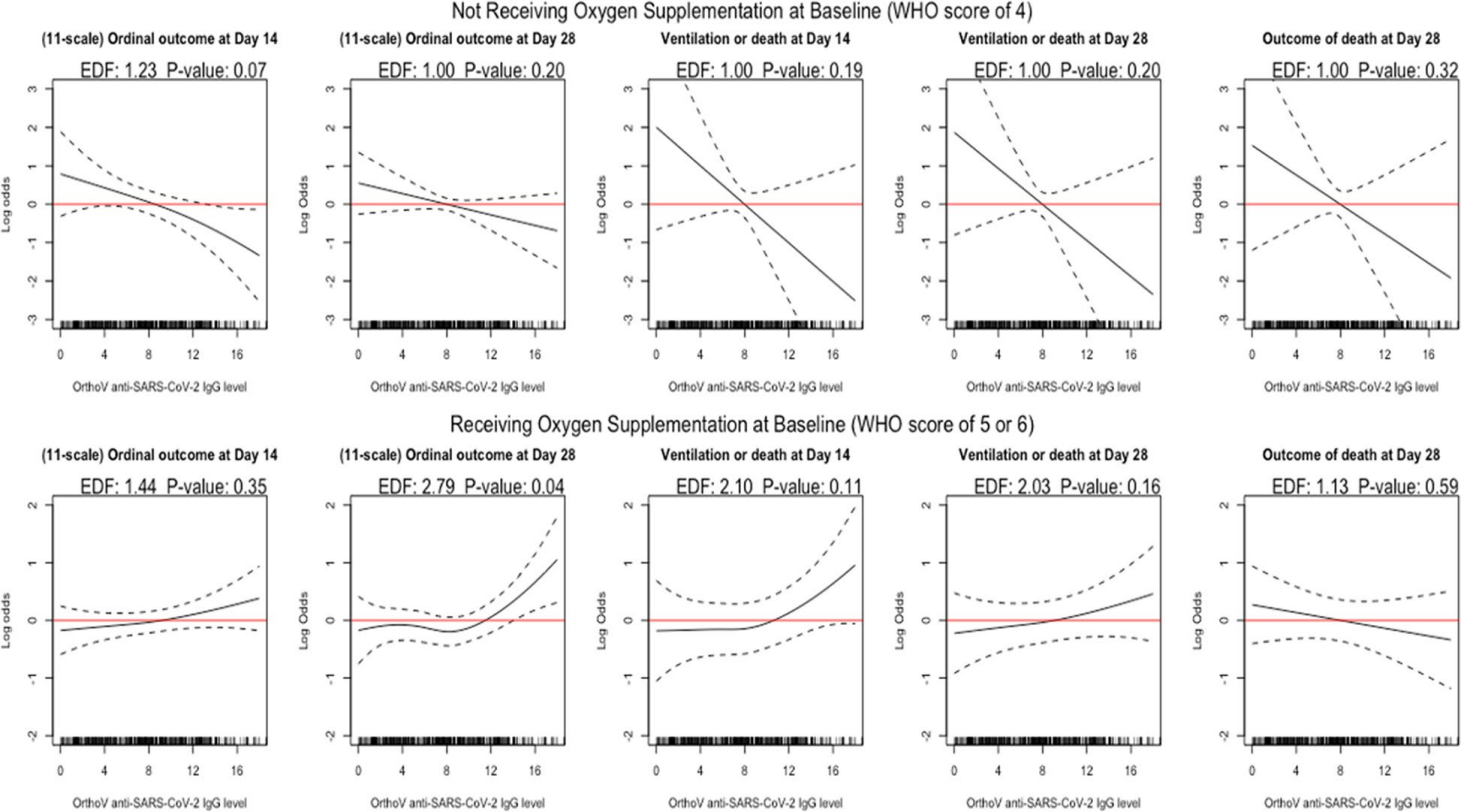
Dose-response curves for five clinical outcomes in CCP-treated patients, stratified by baseline oxygen supplementation status (WHO score of 4, not receiving oxygen supplementation at baseline, in the top row; WHO score of 5 or 6, receiving oxygen supplementation at baseline, in the bottom row), with adjustments for potential confounders (see Table 1). The x-axis represents CCP dose (OrthoV S/Co), and the y-axis shows the log odds of an unfavorable outcome (having a higher 11-point WHO scale at day 14 or day 28, being ventilated or dead at day 14 or day 28, or mortality at day 28) with lower values indicating a more favorable clinical outcome. (95% point-wise confidence bands are overlaid in dashed curves; EDF: the effective degrees of freedom; *P*-value assesses the null hypothesis of no dose-outcome association.)

Of note, TBI is primarily a function of oxygen supplementation status at baseline and certain patient characteristics (Supplementary Table S1). Figure 2 below shows relationships between CCP dose and clinical outcomes for CCP-treated patients (*n* = 576), stratified by recipient baseline oxygen supplementation status.

For participants not receiving oxygen supplementation at baseline (top row in Fig. 2), there was a consistent and linear dose-dependent CCP effect on clinical outcome, although 95% confidence intervals were relatively wide. However, the dose-response association was less clear and nonlinear in patients receiving oxygen supplementation at baseline (bottom row in Fig. 2). Supplementary Materials Figure S2 (page 8) displays the dose-response analyses unstratified by baseline oxygen supplementation status, which also exhibit generally nonlinear dose-response patterns.

**Fig. 3:**
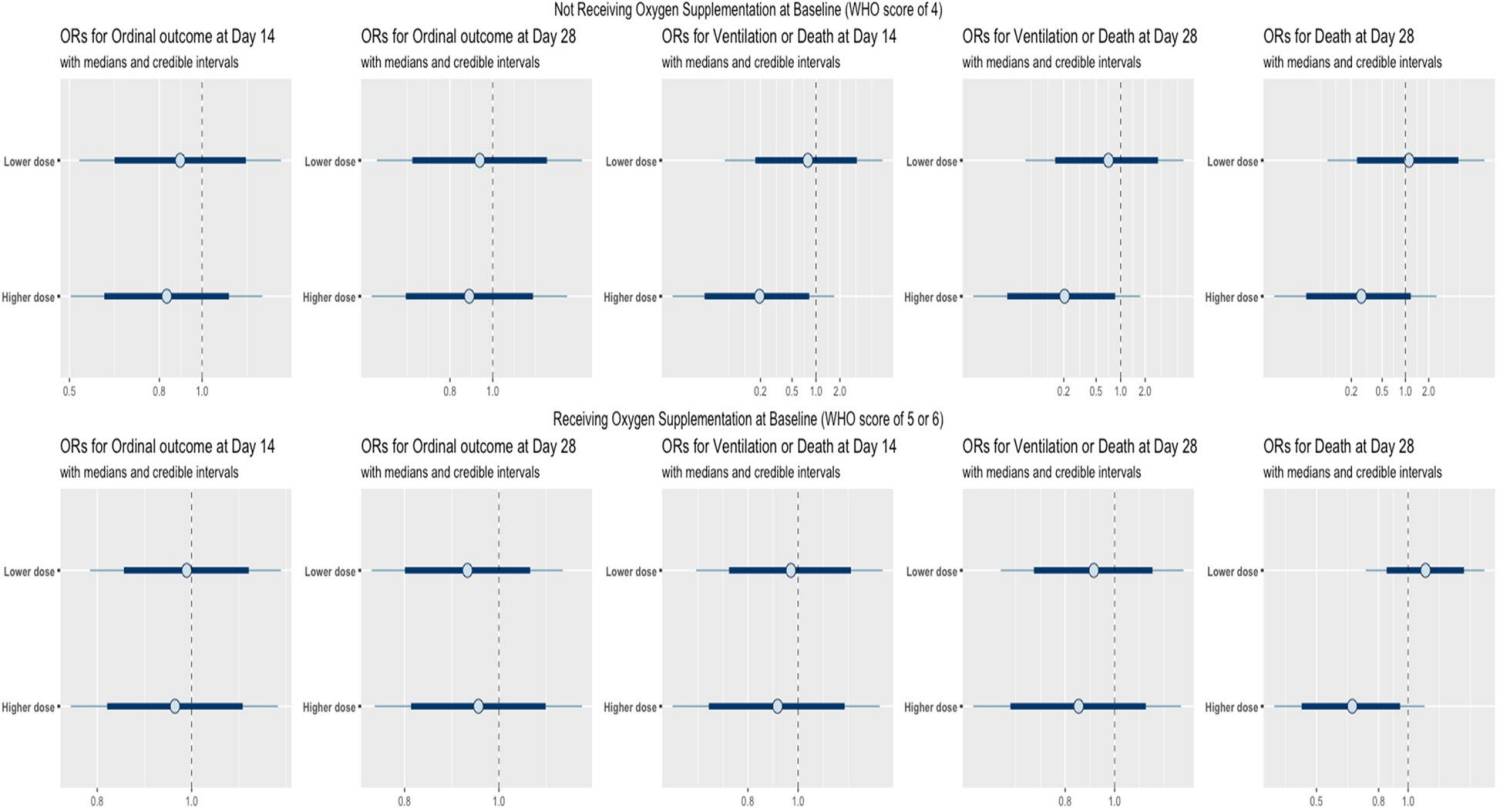
CCP efficacy odds ratios (and 80% and 95% credible intervals) for CCP dose groups (Lower dose: <8 S/Co, and Higher dose: ≥8 S/Co) compared to the control group for each of the 5 outcomes, stratified by baseline oxygen supplementation status (top row: not receiving oxygen supplementation; bottom row: receiving oxygen supplementation), with adjustments for potential confounders (details in Supplementary Section S3.1); the odds ratios < 1 indicate a greater CCP efficacy vs. control

To assess CCP efficacy in comparison to the control group, we examined clinical outcomes in participants receiving lower (< 8 OrthoV S/Co) or higher (≥ 8 OrthoV S/Co) dose CCP, compared to controls (*n* = 1138; see Table 1 for patient characteristics), where an OrthoV S/Co value of 8 corresponded to the observed sample mean in COMPILE (Supplementary Table S2), and raw outcome counts for each dose are reported in Supplementary Materials Table S7. We performed multivariable regression with the dose group as the main regressor with 3 levels (control, lower and higher dose), adjusted for the baseline factors in Table 1 (Supplementary Section S3.1 for detailed specifications of these regression models). Figure 3 shows the (lower or higher) dose-specific CCP efficacy odds ratios (ORs) compared to the control group.

The results in Fig. 3 confirm the dose-outcome associations in Fig. 2. Among participants not receiving baseline oxygen supplementation (top row in Fig. 3), higher-dose CCP (≥ 8) was associated with higher efficacy (/control) for ventilation or death at day 14 (OR = 0.19, 95% credible interval (CrI): [0.02, 1.70], posterior probability Pr(OR < 1) = 0.93, with a higher probability indicating a stronger evidence of CCP efficacy) and for day 28 mortality (OR = 0.27, CrI: [0.02, 2.53], Pr(OR < 1) = 0.87), whereas lower-dose CCP (< 8) showed less efficacy (/control) (for ventilation or death at day 14: OR = 0.79, CrI: [0.07, 6.87], Pr(OR < 1) = 0.58; and for day 28 mortality: OR = 1.11, CrI: [0.10, 10.49], Pr(OR < 1) = 0.46) compared to the higher-dose. For participants receiving oxygen supplementation at baseline (bottom row in Fig. 3), CCP was not effective in either lower or higher dose, although a higher CCP benefit for day 28 mortality was observed for higher-dose CCP (/control) (OR = 0.66, CrI: [0.36, 1.13], Pr(OR < 1) = 0.93) compared to lower-dose CCP (/control) (OR = 1.14, CrI: [0.73, 1.78], Pr(OR < 1) = 0.28). The unstratified analysis, conducted without stratification by baseline oxygen supplementation status (Supplementary Materials Figure S20), also displayed unclear dose-response patterns, except for a superior day 28 mortality benefit of higher-dose CCP (/control) (OR = 0.69, CrI: [0.38, 1.19], Pr(OR < 1) = 0.91) compared to lower-dose CCP (/control) (OR = 1.04, CrI: [0.65, 1.63], Pr(OR < 1) = 0.44).

The stratified analyses by days since symptom onset to treatment initiation (≤ 3 days vs. > 3 days), presented in Supplementary Figures S3 and S21, align with the results stratified by baseline oxygen supplementation (no oxygen supplementation vs. oxygen supplementation) shown in Figs. 2 and 3.

## Discussion & conclusion

Compared to Joyner et al. [1], our inclusion of a control group to perform a dose-outcome analysis enabled a more rigorous comparison of CCP efficacy with different SARS-CoV-2 IgG levels. The results provide new insight into CCP efficacy as a function of WHO-defined COVID-19 clinical status based on oxygen supplementation at presentation [20]. Overall, we observed a positive association between CCP dose and clinical outcome in patients not receiving oxygen supplementation at baseline.

Studies on CCP, ranging from observational case studies to RCTs, have reported variable results in hospitalized COVID-19 patients, with some showing reduced mortality and others showing a lack of efficacy in severely ill patients [22]. However, CCP was effective when administered early in the course of the disease, as reported by Salazar et al. [23] and Libster et al. [11], where high CCP SARS-CoV-2 IgG titers (1:3200 or higher) reduced the risk of severe respiratory disease. More recently, Misset et al. [16] reported a mortality benefit of high-dose CCP in severely ill COVID-19 patients who received mechanical ventilation and were randomized within 48 h of ventilation initiation. Notably, the CCP used in Misset et al. [16] which had a neutralizing titer of at least 1:160 and the highest titer units used in Libster et al. [11]. were likely to contain significantly more SARS-CoV2 IgG and have significantly more neutralizing activity than most of the units used in COMPILE.

Our analysis of COMPILE participants suggests a potential mortality benefit for higher-dose CCP compared to lower-dose CCP, providing RCT evidence to support earlier non-randomized studies indicating the mortality benefit of high-titer CCP [1]. While the CCP dose-outcome association is less clear for patients receiving oxygen supplementation at baseline, there was a consistently linear CCP dose effect on favorable outcomes for patients without oxygen supplementation at baseline, with the OR for death at 28 days for the higher dose group (vs. control) without oxygen supplementation at baseline: 0.27, 95% CrI: [0.02, 2.53], although statistical significance is not reached due to the relatively small sample size for the higher dose group with WHO = 4 at baseline (Table 1). However, this aligns with meta-analyses demonstrating the efficacy of high-titer CCP in early COVID-19 stages [14], and also reinforces historical findings that treatment with convalescent plasma is most effective when administered early in the disease course [24, 25]. The CCP dose-efficacy relationship is further supported by Stadler et al. [26], who reported a significant association between CCP dose and efficacy for preventing hospitalization in outpatients. The benefit of CCP early in the course of COVID-19 aligns with its mechanism of action, which is viral elimination, whereas its lack of efficacy later, in the inflammatory phase when most patients require oxygen supplementation, reflects its inability to reverse established inflammation, particularly in the lungs [17]. The antiviral activity of CCP is highlighted by its mortality benefit in immunocompromised COVID-19 patients who lacked endogenous antibody and do not respond to SARS-CoV-2 vaccines [27]. 

The mechanistic activity of CCP is not fully understood, as factors such as antibody functional activity [28, 29], antibody specificity, isotype, IgG subclass, affinity, and host immune features, including endogenous antibody levels, may influence its effectiveness. Nonetheless, our data support the conclusion that CCP efficacy early in the course of COVID-19 is likely to stem from its ability to neutralize SARS-CoV-2. SARS-CoV-2 IgG levels were highly correlated with neutralizing titers [30]. Although comprehensive studies of the entire profile of CCP antibodies along with host characteristics including the endogenous immune response, are needed to gain further insight into CCP efficacy in the inflammatory stage of disease [31], our data show that CCP with high levels of antibody is likely to confer a benefit in patients who do not require oxygen supplementation. The nonlinear dose-response curves in patients requiring oxygen supplementation, who are likely past the viral replication phase and have likely entered a phase where inflammation drives tissue damage, may reflect various patient and CCP factors, including the need for higher titer CCP and perhaps prozone-like effects [24, 32, 33]. Further analyses and research are warranted to better understand the mechanisms through which CCP antibody levels affect the effectiveness of CCP. This research is important because convalescent plasma (CP) may be the only therapy available in future infectious disease outbreaks [34] as well as the least costly treatment in settings where anti-microbial drugs are not available [35]. An important element of future research should be the use of standardized platforms to vet CP activity based on antibody activity against the relevant agent and ensure that the highest amount of active antibody is administered.

## Strengths & limitations

A major strength of our study is its RCT design, which provides robust evidence that higher dose CCP is likely beneficial for hospitalized patients not requiring oxygen supplementation at enrollment by inclusion of a control group. However, several limitations should be noted.

More precise measurements of the CCP ‘dose,’ e.g., quantitative antibody assays, would have been preferable. For a limited number of samples among participants from CONTAIN [4], an RCT included in COMPILE (*n* = 135), we compared OrthoV measurements to quantitative measures of CCP (spike protein-IgG half-maximal effective concentrations (EC50) and CCP neutralizing titers). Consistent with Farnsworth et al. [36], who reported a linear relationship between OrthoV and neutralizing antibody titers, there was a clear linear association between the OrthoV measurement and these quantitative measures (Supplementary Materials S4.1).

We analyzed the relationship between clinical outcome and CCP dose without inclusion of post-CCP participant antibody levels as COMPILE did not collect this data. We acknowledge that pre-treatment antibody levels may have influenced our results. Although our data showed that baseline seropositive participants, in comparison to seronegative participants, were more likely to survive with a day 28 mortality odds ratio of 0.73 (95% CI: [0.56, 1.12]), serostatus at baseline was also significantly associated with baseline disease severity (*p*-value < 0.01) as was enrollment quarter (*p*-value < 0.01) (Supplementary Materials S4.2), potentially confounding the association. However, there was no significant association between pre-treatment serostatus and CCP (higher and lower) dose groups (Table 1) and we used pre-treatment serostatus as an adjusting variable in our analyses. Furthermore, it is likely that all the participant antibody measured at enrollment was endogenous, since the COMPILE study completed enrollment in March 2021, which is when vaccination was first rolled out to high-risk older patients. While SARS-CoV-2 variants could have affected CCP efficacy over time, the effects of SARS-CoV-2 variants and the collection date of CCP on our results are unknown.

Although patient allocation to CCP or control conditions was randomized, CCP dose allocation lacked predefined rules. If related to patient characteristics, this could introduce unmeasured confounding factors, and the exclusion of patients with missing antibody measures could introduce bias. Yet, at the time of CCP administration, CCP antibody level was unknown for all samples. Additionally, the absence of plasma samples for measurement by the OrthoV assay was not due to individual patient characteristics, which mitigates concerns about CCP dose allocation and selection bias. The study has limitations related to heterogeneity among participating RCTs, as it combines data from multiple trials with diverse patient populations and treatment protocols. Retrospective measurement of CCP antibody levels may not also fully represent antibody levels in administered CCP. Nonetheless, we mitigated potential biases by adjusting for the participating RCTs as a covariate to account for heterogeneity among trials, performing sensitivity analyses to account for missing antibody measures (Supplementary Section S2.3), and exploring CCP dose interactions with patient baseline covariates (Supplementary Sections S2.2 and S3.2). Future studies could more thoroughly investigate CCP dose interactions with antiviral treatments and additional patient characteristics. This will align with Sheiner’s [37] emphasis on exploratory analysis for quantitatively assessing patient benefit based on treatment regimen, concomitant therapies, and patient characteristics in clinical treatment development. Our findings offer insights into the relationship between CCP dose and treatment outcomes, particularly regarding baseline oxygen supplementation status. This adds a layer to a potentially personalized CP treatment roadmap, with implications for further research and clinical practice.

### Supplementary Information

Supplementary Material 1.

## Data Availability

The data that support the findings of this study are available from COMPILE (which is a consortium contributed by individual randomized controlled trials and has contracts with these contributing randomized controlled trials that govern data use) but restrictions apply to the availability of these data, which were used under license for the current study, and so are not publicly available. Data are, however, available from the corresponding author upon reasonable request and with permission of COMPILE.
