# Supplementary material for: Association between COVID-19 convalescent plasma antibody levels and COVID-19 outcomes stratified by clinical status at presentation
Source: BMC Infect Dis. 2024 Jun 26;24:639. doi: 10.1186/s12879-024-09529-0 (PMC11201301; doi:10.1186/s12879-024-09529-0)
Supplement: Supplementary file 1 — Supplementary Material 1. [file 12879_2024_9529_MOESM1_ESM.pdf]

Association between COVID-19 Convalescent Plasma Antibody Levels and COVID-19 Outcomes Stratified by Clinical Status at Presentation

Supplement

Section S1. Study Design – p5
Section S2. Continuous dose-response analysis – p7
S2.1. Main continuous dose-response analysis – p7
S2.2. Continuous dose-by-covariate interaction analysis – p10
S2.3. Sensitivity analysis – p18
Section S3. Antibody group analysis – p24
S3.1. Main dose group analysis – p24
S3.2. Dose group-by-covariates interaction analysis – p33
Section S4. Additional information – p38
S4.1. Correlations of OrthoV with spike-IgG EC50 and neutralizing titer – p38
S4.2. Correlations of recipients' baseline seropositive status with other variables – p39
S4.3. Uniform testing of antibody titers on the OrthoV platform in COMPILE RCTs – p40

Figure S1. Full WHO (World Health Organization) 11-point COVID-19 patient status scale[1]. This WHO 11-scale ordinal outcome was measured at day 14 and day 28 post-treatment as well as at baseline. The binary outcome of ventilation or death corresponds to the indicator of WHO score ≥ 7.

Score	Descriptor
0	Uninfected; no viral RNA detected
1	Asymptomatic; viral RNA detected
2	Symptomatic: Independent
3	Symptomatic: assistance needed
4	Hospitalized; no oxygen therapy
5	Hospitalized; oxygen by mask or nasal prongs
6	Hospitalized; oxygen by NIV or High flow
7	Intubation & Mechanical ventilation, pO2/FIO2>/=150 or SpO2/ FIO2>/=200
8	Mechanical ventilation pO2/FIO2<150 (SpO2/FIO2<200) or vasopressors
9	Mechanical ventilation pO2/FIO2<150 and vasopressors, dialysis or ECMO
10	Death

[1] WHO Working Group on the Clinical Characterisation and Management of COVID-19 infection (2020). "A minimal common outcome measure set for COVID-19 clinical research." Lancet Infect Dis 20(8): e192-e197.

Table S1. Coefficients of the linear combination for baseline patient characteristics in the treatment-benefit-index (TBI)[2].

Baseline characteristics	Coefficients** (95% bootstrap CIs)
Intercept	0.36 (0.08, 0.65)
Oxygen support by mask or nasal prongs* (1/0)	-0.24 (-0.45, -0.04)
Oxygen support by high flow* (1/0)	-0.17 (-0.48, 0.13)
Oxygen support by mask or nasal prongs* & age ≥67 (1/0)	-0.04 (-0.26, 0.17)
Oxygen support by high flow* & age ≥67 (1/0)	-0.19 (-0.60, 0.22)
Blood type A or AB (1/0)	0.14 (-0.07, 0.36)
Cardiovascular Disease (1/0)	0.19 (0.00, 0.38)
Comorbid Diabetes Mellitus & Pulmonary Disease (1/0)	0.30 (-0.12, 0.72)

*The reference level: hospitalized but no oxygen therapy required
**TBI is calculated as the sum of the intercept (=0.36) and the product of patient characteristic indicators (1/0) and their corresponding coefficients.

[2] Park, H., et al. (2022). "Development and Validation of a Treatment Benefit Index to Identify Hospitalized Patients With COVID-19 Who May Benefit From Convalescent Plasma." JAMA Netw Open 5(1): e2147375

Table S2. Summary of CCP OrthoV antibody measures in the COMPILE[3] study by individual RCTs.

RCTs	Total enrollment **(n)**	Control **(n)**	CCP **(n)**	CCP antibody measures **(n)**	CCP missing antibody measures **(n)**	"OrthoV" signal-to-cutoff (S/Co) ratio **Mean (SD)**	"OrthoV" signal-to-cutoff (S/Co) ratio **Range**
NYC	941	473	468	141	327	5.38 (3.98)	[0.06, 17.5]
UPenn	80	39	41	39	2	5.91 (3.49)	[1.26, 14.4]
Spain	350	171	179	175	4	8.11 (4.02)	[0.00, 15.4]
UCSF	34	18	16	16	0	17.75 (2.78)	[14.6, 22.56]
Belgium	477	163	314	151	163	8.49 (3.31)	[0.23, 16.3]
Brazil	34	15	19	17	2	7.48 (4.55)	[1.53, 12.6]
Netherlands	72	35	37	37	0	12.86 (5.20)	[2.28, 18.7]
India	381	224	157	0	157	NA	NA
Total	2369	1138	1231	576	655	7.94 (4.59)	[0.00, 22.56]

[3] Troxel, A. B., et al. (2022). "Association of Convalescent Plasma Treatment With Clinical Status in Patients Hospitalized With COVID-19: A Meta-analysis." JAMA Netw Open 5(1): e2147331.

Section S1. Study Design

To assess the association between SARS-CoV-2 IgG levels (antibody levels) in COVID-19 convalescent plasma (CCP) and its therapeutic effectiveness, we used data from the COMPILE[4] study, a meta-analysis of pooled individual patient data from 8 RCTs that evaluated CCP vs control in adults hospitalized for COVID-19. The COMPILE inclusion criteria define the study population as hospitalized patients with confirmed COVID-19 who were not on a mechanical ventilator at the time of randomization.

The analysis involved n =1714 COVID-19 hospitalized patients not on mechanical ventilators at the time of randomization, including 1138 control patients and 576 CCP-treated patients with available CCP antibody measurement information. Baseline characteristics of these patients, stratified by two (lower and higher) anti-SARS-CoV-2 IgG antibody level groups and the non-CCP control group, as well as the group of patients with missing CCP antibody measures, are summarized in **Table 1** of the main manuscript.

The study outcomes were the World Health Organization (WHO) 11-point ordinal COVID-19 clinical status scale (see Figure S1) and its two derivative binary outcomes (i.e., WHO score of 7-10, indicating mechanical ventilation to death, and WHO score of 10, indicating death), assessed at 14 ± 1 days (hereafter, day 14) and 28 ± 2 days (hereafter, day 28) after randomization. The co-primary outcomes of COMPILE were the binary indicator of WHO score of 7-10 (indicating mechanical ventilation to death) and the WHO 11-point ordinal scale at day 14 post-treatment.

The SARS-CoV-2 spike-binding IgG levels in the donor's plasma were measured retrospectively, using CCP donor sera obtained at the time of donation (on samples that the blood banks retained from each donor sample) or the administered convalescent plasma (in cases from the NYC RCT). The measurements were made semi-quantitatively using the Ortho Clinical Diagnostics VITROS® XT7600 Integrated System Anti-SARS-CoV-2 assay (OrthoV) according to the manufacturer's protocol, which was consistent across all the COMPILE RCTs.

The OrthoV platform was previously used to retrospectively determine SARS-CoV-2 IgG levels in donor sera from convalescent plasma units in the Mayo Clinic Expanded Access Program study[5]. In August 2020, high titer convalescent plasma was authorized for emergency use for hospitalized patients with COVID-19 by the US FDA[6]. [Of note, this recommendation has been changed in February 2021 to limit the use of CCP with high titers to the treatment of COVID-19 in outpatients or inpatients with immunosuppressive disease or receiving immunosuppressive treatment.] OrthoV was also the first platform authorized by the FDA for labeling convalescent plasma units as 'high titer'[7].

We used the SARS-CoV-2 IgG signal-to-cutoff ratio (S/Co) as a continuous measure of CCP antibody amount (OrthoV dose). We also conducted a dose-level group analysis to explore potential heterogeneous CCP effects by dose levels vs. control. We divided the doses into two groups: lower dose with S/Co <8 and higher dose S/Co ≥ 8, where the S/Co value of 8 roughly corresponds to the observed mean of the OrthoV measure in this study.

---

[4] Troxel, A. B., et al. (2022). "Association of Convalescent Plasma Treatment With Clinical Status in Patients Hospitalized With COVID-19: A Meta-analysis." JAMA Netw Open 5(1): e2147331.
[5] Joyner, M. J., et al. (2021). "Convalescent Plasma Antibody Levels and the Risk of Death from Covid-19." N Engl J Med 384(11): 1015-1027.
[6] US Food, Drug Administration, et al. "Recommendations for investigational covid-19 convalescent plasma." Food and Drug Administration, 2020
[7] Hinton, D. M. Letter from the US FDA https://www.fda.gov/media/141477/download

In some COMPILE participating RCTs (UPenn, Belgium and Netherlands), patients received multiple units of CCP. To handle this scenario, we took the average of the SARS-CoV-2 IgG levels measured using the OrthoV platform for those patients.

As the amount of antibody in the CCP (dose) was not intentionally randomized to the CCP recipients at the time of the treatment, we employed multi-variable regression to adjust for potential confounding effects in our analyses. The main exposure was the amount of antibody (dose), treated either as continuous or categorized. In the multivariable regression models, we incorporated RCT-specific intercepts to account for heterogeneity between different trials, and included a set of "**expanded covariates**" and "**concomitant medications**" which might have impacted the study outcomes as adjusting variables.

The "**expanded covariate set**" includes the baseline WHO score (representing the oxygen support status at baseline), enrollment calendar quarter, age, sex, patient weight, days since symptoms onset, blood type, systolic blood pressure, baseline serostatus, binary indicators for history of asthma, diabetes, pulmonary, and cardiovascular diseases, and the participating RCT IDs (Table 1).

The "**concomitant medication set**" includes the indicators for the use of concomitant medications, including hydroxychloroquine, antibacterial, antiviral, remdesivir, anti-inflammatory, steroids, antithrombotic, antiplatelet agents and anticoagulant agents at the time of randomization (Table 1).

The analysis was conducted in a way that was consistent with the main COMPILE study[8]. For example, the way the baseline variables were coded (e.g., age is categorized into 3 groups, <50, 50-65, and >65) and the binary outcome variables (the indicators of mechanical ventilation or death and of death) are defined was consistent with the main COMPILE study. Throughout the study, we used logistic regression models to analyze binary outcomes, and cumulative logit proportion odds models to analyze ordinal outcomes (the WHO 11-point ordinal COVID-19 clinical status scales).

Throughout, to address the issue of sporadic missingness in baseline covariates when we adjust for the expanded covariate and concomitant medication sets, we used multiple imputation (MI)[9] to impute the covariates. We did not impute the outcomes nor the CCP dose information, but only missing baseline covariates included in the adjusting variable set. Specifically, we imputed the missing baseline covariates m=100 times using only the baseline information by predictive-mean-matching, and the analysis results were subsequently combined across these 100 multiply-imputed datasets (details provided in Sections S2.1 and S3.1).

---

[8] Troxel, A. B., et al. (2022). "Association of Convalescent Plasma Treatment With Clinical Status in Patients Hospitalized With COVID-19: A Meta-analysis." JAMA Netw Open 5(1): e2147331.
[9] van Buuren S, Groothuis-Oudshoorn K (2011). "mice: Multivariate Imputation by Chained Equations in R." Journal of Statistical Software, 45(3), 1-67.

Section S2. Continuous dose-response analysis

S2.1. Main continuous dose-response analysis

The continuous dose-response analysis involved the patients from the CCP treatment arm who received plasma confirmed to contain SARS-CoV-2 IgG and the donor's antibody level or antibody level in the administered CCP available (n=576). Among the 1231 CCP-treated patients in COMPILE, 576 had retrospectively measured OrthoV S/Co CCP antibody doses, reported in **Table S2**.

To fit the dose-response relationship and investigate the potential nonlinear association between the outcomes and the CCP dose, we used restricted cubic-spline representations of the continuous dose effect on each outcome, adjusting for the "**expanded covariate**" and "**concomitant medication**" sets, within a generalized additive modeling (GAM) framework. The roughness parameter associated with the restricted cubic-spline representation of the dose-response curve is determined based on the restricted maximum likelihood (REML) criterion. To test for possibly nonlinear dose-outcome associations, we conducted a Chi-squared test for assessing the statistical significance[10] of the estimated curves, as implemented in the R package "mgcv"[11].

Figure 2 of the main manuscript displays the resulting dose-response curves for five clinical outcomes (day 14 and 28 WHO ordinal clinical scales and indicators of mechanical ventilation or death, and day 28 mortality). The curves show the relationship between antibody dose and the log odds of unfavorable outcomes (where lower values are clinically desirable) among the CCP-treated patients, stratified by the baseline oxygen support requirement status.

In Figure 2 of the main manuscript, the effective degrees of freedom (EDF) quantify the flexibility of the restricted cubic spline fit for the dose main effect (for example, an EDF of 1 indicates that the data shows no nonlinear association, suggesting a linear relationship), and the associated P-value on the null hypothesis of no association is a measure of the strength of this dose-outcome association. Of note, using the median p-value (MPV) rule[12], the significance test results (EDF and P-value) for possibly nonlinear dose-outcome association are combined across the m=100 imputed datasets and reported in each panel's subtitle, whereas the dose-response curves are drawn from one representative dataset randomly selected from the imputed datasets.

The unstratified dose-response analyses, not stratified by baseline oxygen supplementation status, are presented in **Figure S2**, which shows unclear, nonlinear dose-response patterns.

Mirroring the analyses conducted with stratification by baseline oxygen support status, similar analyses were conducted by stratifying days since symptom onset to treatment initiation (≤3 vs. >3 days), and the resulting dose-response curves are displayed in **Figure S3**.

---

[10] Wood, S. N. (2013). "On p-values for smooth components of an extended generalized additive model." Biometrika, Volume 100, Issue 1, March 2013, pp. 221–228.
[11] Wood, S. N. (2011). "Fast stable restricted maximum likelihood and marginal likelihood estimation of semiparametric generalized linear models." Journal of the Royal Statistical Society (B), Vol. 73, Issue 1, pp. 3–36.
[12] Bolt MA, MaWhinney S, Pattee JW, Erlandson KM, Badesch DB, Peterson RA. "Inference following multiple imputation for generalized additive models: an investigation of the median p-value rule with applications to the Pulmonary Hypertension Association Registry and Colorado COVID-19 hospitalization data." BMC Med Res Methodol. 2022 May 21;22(1):148

Figure S2. The dose-response curves for five clinical outcomes showing the relationship between antibody dose and the log odds of unfavorable outcomes among the CCP-treated patients, **not stratified by the baseline oxygen supplementation status**. The horizontal axis represents the antibody dose measured on OrthoV (S/Co), and the vertical axis represents the log odds of an unfavorable outcome with lower values clinically desirable.

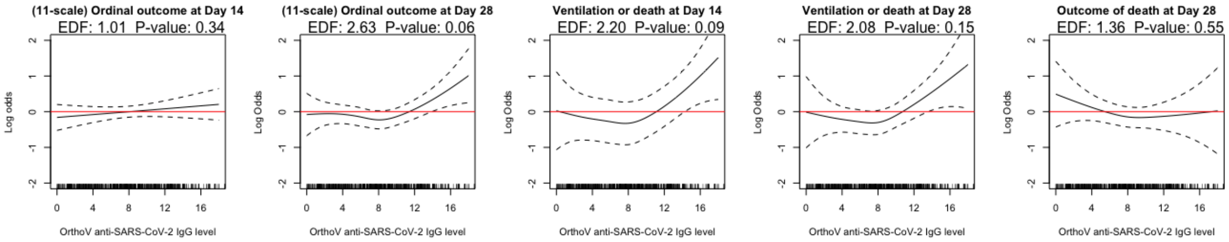

Figure S3. The dose-response curves for five clinical outcomes showing the relationship between antibody dose and the log odds of unfavorable outcomes among the CCP-treated patients, stratified by the *time since symptom onset to treatment initiation* (≤ 3 days in the top row, vs. > 3 days in the bottom row), adjusting for the expanded covariate and concomitant medication sets. The horizontal axis represents the antibody dose measured on OrthoV (S/Co), and the vertical axis represents the log odds of an unfavorable outcome with lower values clinically desirable.

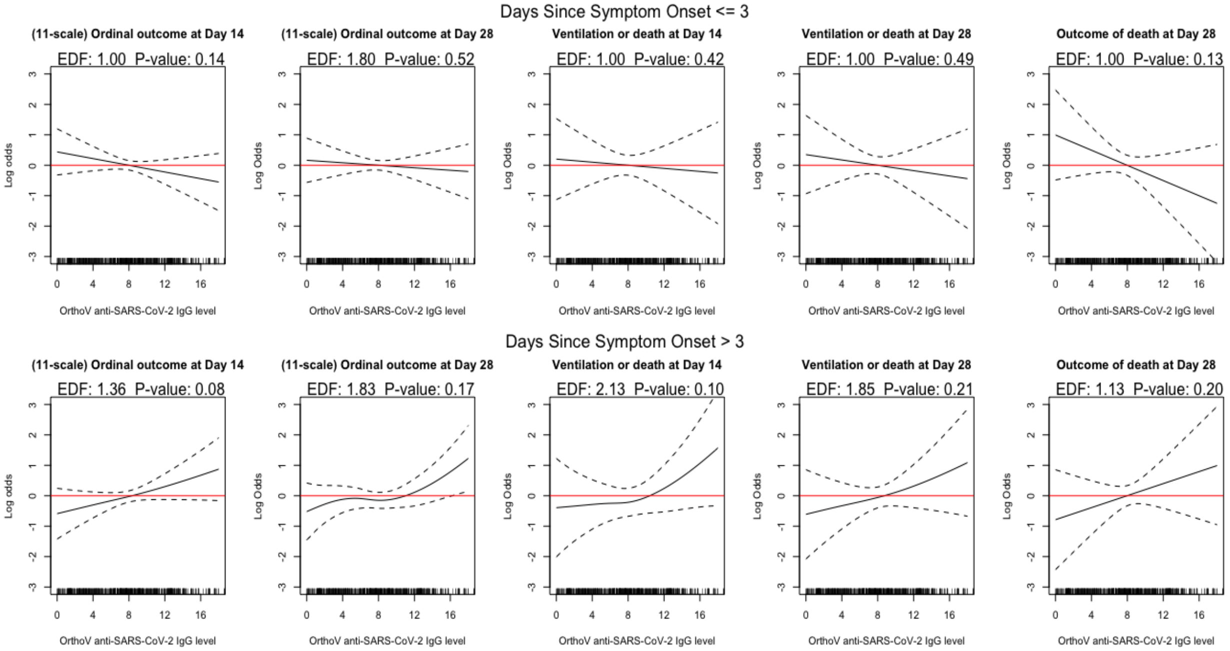

S2.2. Continuous dose-by-covariate interaction analysis

In addition to the primary dose-outcome analysis by baseline oxygen support status (Figure 2 in the main manuscript), we explored variations in the dose-outcome association with other baseline factors.

To capture potential nonlinear differences in dose effects across baseline covariates, we included the main dose effect (represented as a restricted cubic spline), the effect for each potential moderating factor, and the dose-by-factor interaction effect (also represented as a restricted cubic spline for each level of the potential moderating factor), adjusting for the expanded covariate and concomitant medication sets. Then, using Wood's Chi-squared test[13], we tested the statistical significance of each baseline factor's level-specific dose-response curve, to assess whether each baseline covariate's level-specific dose-response curve is significantly different from its reference level dose-response curve.

In **Table S3**, we summarize the results of Chi-squared tests for level-specific smooth terms on each of the 5 outcomes, reporting median EDFs and p-values from 100 multiply-imputed datasets.

In **Figures S4-S18**, for baseline factors showing significant dose-outcome association variations across their levels (p-value < 0.1) with respect to the primary binary outcome (day 14 post-treatment ventilation or death), we present their factor level-specific dose-response curves, in addition those for patient age, sex and baseline symptom severity.

[13] Wood, S. N. (2013). "On p-values for smooth components of an extended generalized additive model." Biometrika, Volume 100, Issue 1, March 2013, Pages 221–228

Table S3. Results of interaction analyses for CCP dose and baseline covariates. For each baseline covariate, we investigated the potential (possibly nonlinear) dose-by-covariate interaction effects on the five outcomes, while adjusting for the expanded covariate set and the concomitant medication set, based on generalized additive cumulative logistic or logistic regression models. We tested for the significance of the moderating effect of each baseline covariate on the dose-outcome relationships, by examining whether each baseline covariate's level-specific dose-response curve is significantly different from its reference level dose-response curve. We report the median values of the effective degrees of freedom (EDF) (quantifying the degrees of nonlinearity) and the P-values (associated with the null hypothesis of no difference from the reference-level curve) computed from the 100 imputed datasets.

	11-scale ordinal at day 14		11-scale ordinal at day 28		Ventilation or death at day 14		Ventilation or death at day 28		Death at day 28	
	EDF	P-value	EDF	P-value	EDF	P-value	EDF	P-value	EDF	P-value
RCT: NYC (ref)										
RCT: UPenn	1	0.451	1.00	0.180	1	0.348	1	0.166	1.00	0.192
RCT: Spain	1	0.812	1	0.447	1	0.709	1	0.306	1.00	0.467
RCT: UCSF	1.52	0.266	1	0.737	1.98	1	1.98	1	1.98	1
RCT: Belgium	1.60	0.503	2.02	0.324	1.00	**0.097**	1.02	0.121	1.59	0.417
RCT: Brazil	1	**0.009**	1	0.112	2.93	1	2.93	1	1.02	0.611
RCT: Netherland	2.65	**0.010**	2.21	**0.057**	1.16	0.419	1.00	**0.099**	1.60	0.506
Age <=50 (ref)										
Age (50,65]	2.29	0.184	1.00	0.247	2.13	0.428	2.28	0.375	2.59	0.133
Age > 65	2.18	0.454	1.65	0.234	1	0.495	1	0.460	1	0.298
Sex Female	1.00	0.920	1.00	0.334	1	0.944	1	0.916	1	0.818
Blood type O (ref)										
Blood type A	1	0.659	1	0.196	1	**0.034**	1	**0.079**	1	0.173
Blood type B	1	0.237	1	**0.026**	1	**0.001**	1	**0.026**	1	0.275
Blood type AB	1	0.907	2.67	0.118	2.24	0.782	2.09	0.667	2.24	0.905
Systolic BP (>128)	1.00	0.925	1.00	0.565	1	**0.030**	1	0.245	1.13	0.478
Weight (>90kg)	2.88	0.243	1.00	0.443	3.91	**0.014**	2.01	0.491	3.71	**0.099**
Asthma	2.00	0.192	1.01	0.540	2.09	0.285	1.90	0.562	1	0.433
Diabetes	1	0.957	1.00	0.213	2.71	0.462	3.78	0.177	1	0.767
Pulmonary disease	1.00	**0.055**	1.00	**0.012**	1	**0.082**	1	0.142	1.00	0.267
Cardiovascular disease	1	0.218	1.00	0.859	1	0.372	1	0.909	1	**0.066**
Days onset 0-3 (ref)										
Days onset 4-6	1	0.219	1	0.339	1	0.137	1.00	0.196	1	0.226
Days onset 7-10	2.41	**0.016**	2.41	**0.049**	2.54	**0.020**	2.31	**0.072**	2.37	0.139
Days onset 11-14	1.00	**0.062**	1.67	0.163	1.00	0.315	1.93	1	1.93	1
Days onset >14	1.23	0.501	1.00	0.108	1	0.113	1	**0.092**	1	**0.054**
Apr-June 2020 (ref)										
July-Sept 2020	2.67	0.489	1.00	0.449	1	0.113	1	**0.006**	1	**0.023**
Oct-Dec 2020	1	0.958	1	0.959	1	0.890	1	0.214	1	0.672
Jan-Mar 2021	1	0.406	1	0.974	1.00	0.666	1	**0.037**	1	0.211
Antiplatelet	1.79	0.120	2.35	0.212	1.00	**0.036**	1	0.114	3.98	**0.003**
Anticoagulant	1.00	0.719	1.00	0.460	2.4	**0.089**	2.19	0.344	2.13	0.429
Renal therapy	1.02	0.607	1.33	0.668	1	0.786	1	0.811	1	0.810
Serostatus (+)	2.04	0.221	2.20	0.159	1	0.526	1	0.889	1	0.509
HCQ sulfate	1.00	0.202	1.00	0.306	1	0.264	1	1	1	1
Antibacterial	1.00	0.520	1.46	0.207	1	0.945	1	0.219	1	0.379
Antiviral	1.00	**0.024**	1.00	0.103	1	0.415	1	0.308	1	0.765
Remdesivir	1.01	0.530	1.00	0.544	1.02	0.831	1	0.852	1	0.894
Anti-inflammatory	1.00	0.338	1.00	0.743	1	0.238	1	0.131	1	0.498
Steroids	1.00	0.436	1.00	0.293	1.05	0.388	1	0.626	1	0.249
Antithrombosis	1.00	0.426	1.06	0.816	2.09	0.390	2.51	0.264	1.78	0.585
WHO = 4 (ref)										
WHO = 5	2.52	**0.017**	2.48	**0.049**	2.43	0.129	1	0.136	1	0.266
WHO = 6	1	0.283	1.00	0.605	1	0.634	1	0.644	1.43	0.689

Figure S4. The participating RCT-specific dose-response association for the binary outcome of ventilation or death at day 14. Of note, the NYC RCT (represented in the first panel) only enrolled patients with baseline WHO= 5 or 6 (i.e., patients receiving oxygen support at baseline) and the increasing trend (increasing odds of a bad outcome over dose) in this panel "RCT:NYC" is consistent with the dose-response results reported in Figure 2 (bottom row) of the main manuscript.

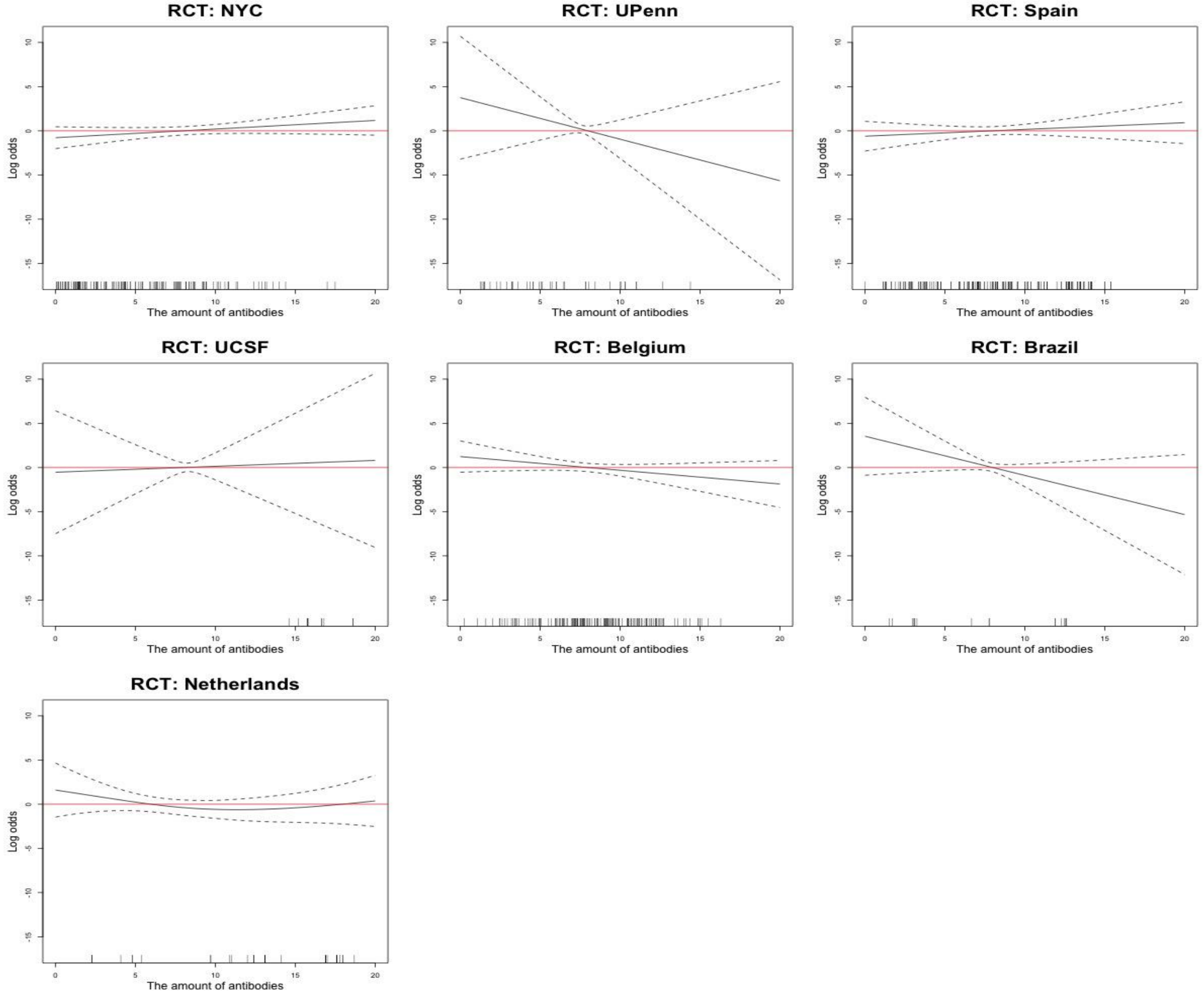

Figure S5. The age group-specific dose-response association for the binary outcome of ventilation or death at day 14.

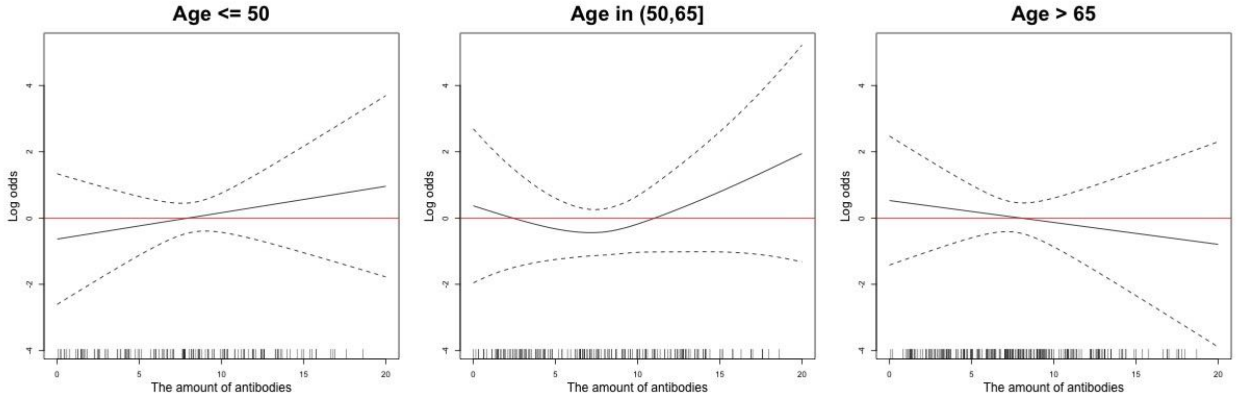

Figure S6. Sex-specific dose-response association for the binary outcome of ventilation or death at day 14.

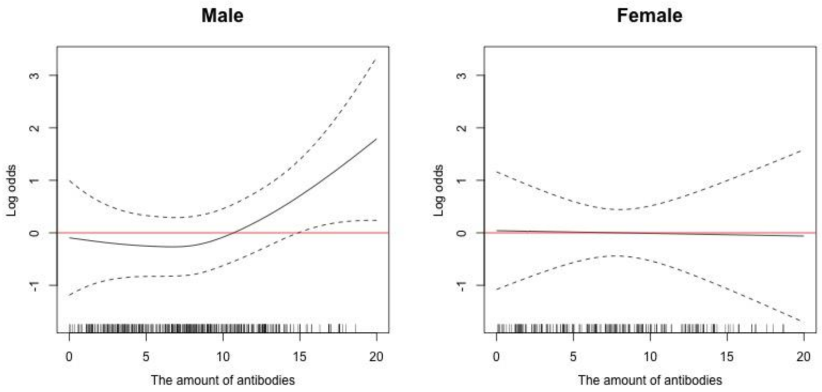

Figure S7. The baseline WHO score (= 4, 5 and 6) group-specific dose-response association for the binary outcome of ventilation or death at day 14.

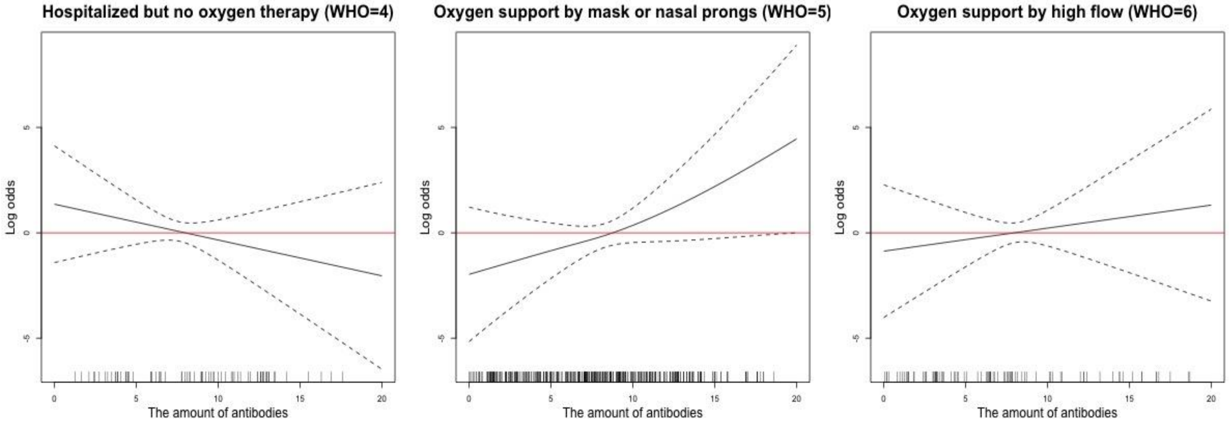

Figure S8. The baseline systolic blood pressure (BP) group-specific dose-response association for the binary outcome of ventilation or death at day 14.

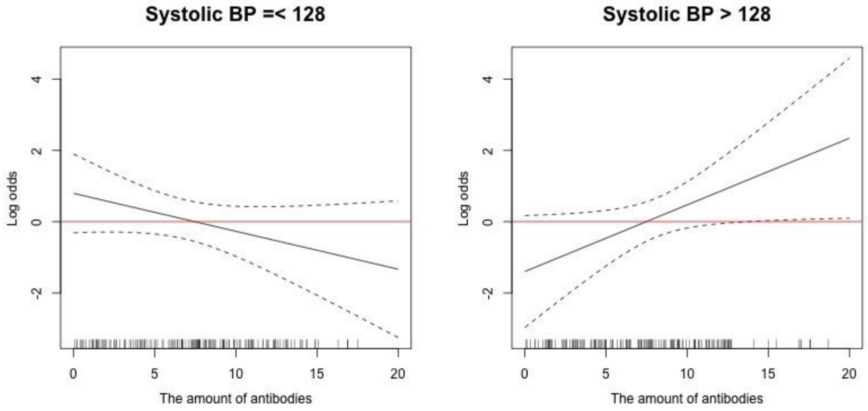

Figure S9. The blood group (O, A, B, AB)-specific dose-response association for the binary outcome of ventilation or death at day 14.

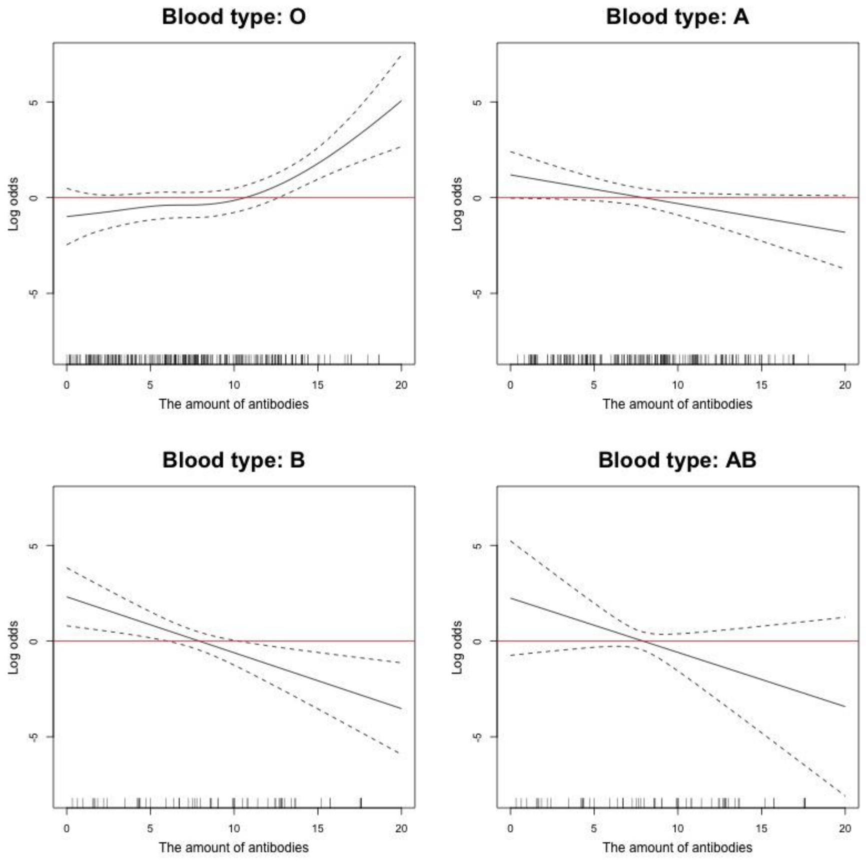

Figure S10. The history of asthma group-specific dose-response association for the binary outcome of ventilation or death at day 14.

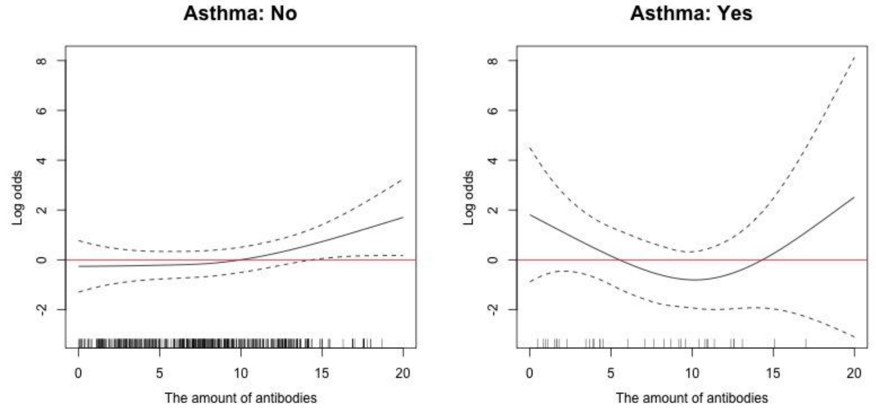

Figure S11. The history of diabetes group-specific dose-response association for the binary outcome of ventilation or death at day 14.

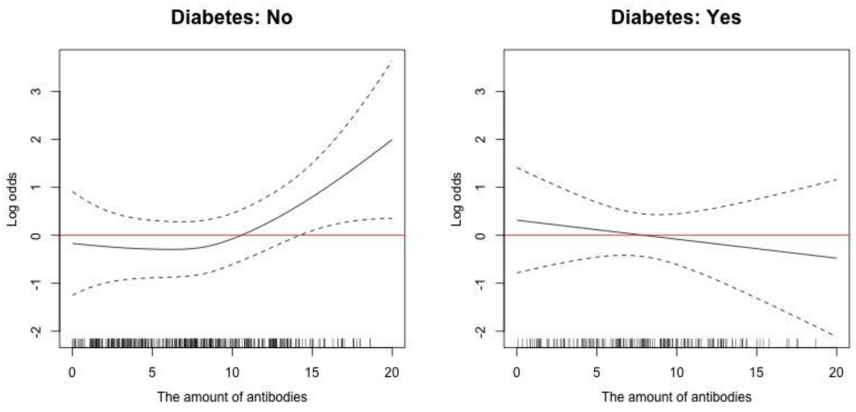

Figure S12. The history of pulmonary disease group-specific dose-response association for the binary outcome of ventilation or death at day 14.

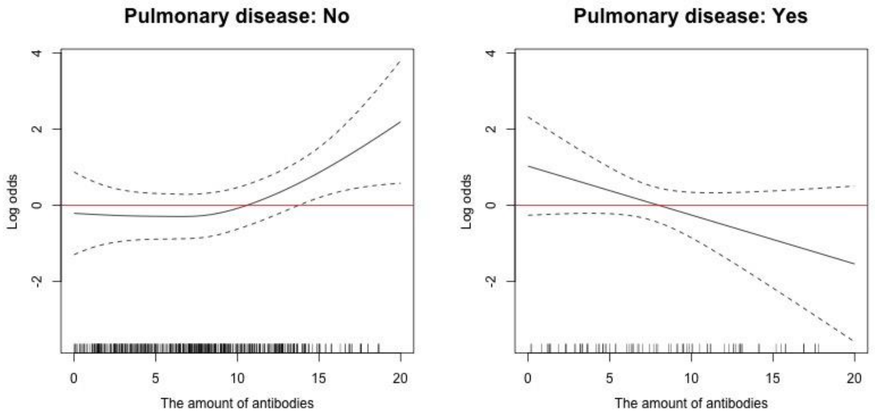

Figure S13. The history of cardiovascular disease group-specific dose-response association for the binary outcome of ventilation or death at day 14.

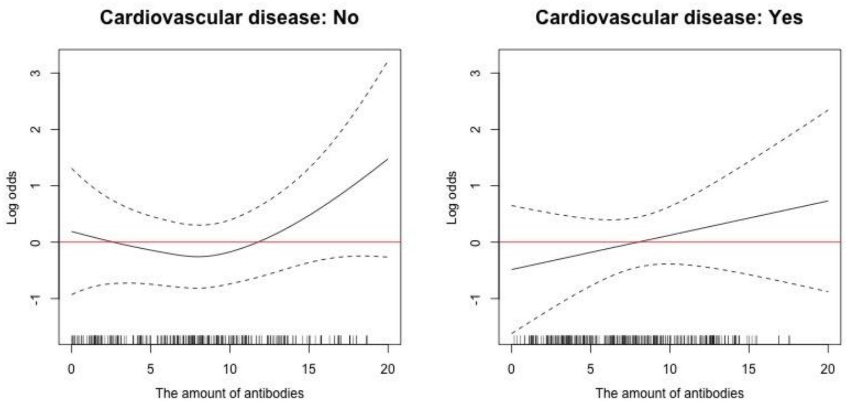

Figure S14. The weight ($\leq$ 90 kg vs. $>$90 kg) group-specific association between the binary outcome of ventilation or worse at day 14 and the CCP dose of antibodies.

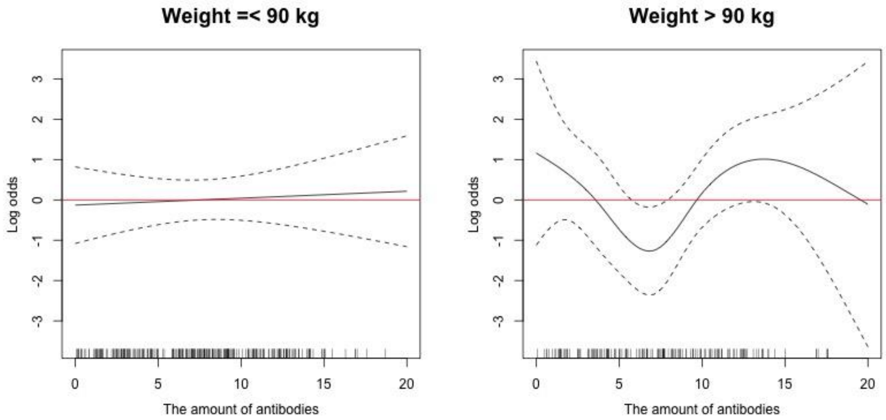

Figure S15. The days since symptoms onset group-specific dose-response association for the binary outcome of ventilation or death at day 14.

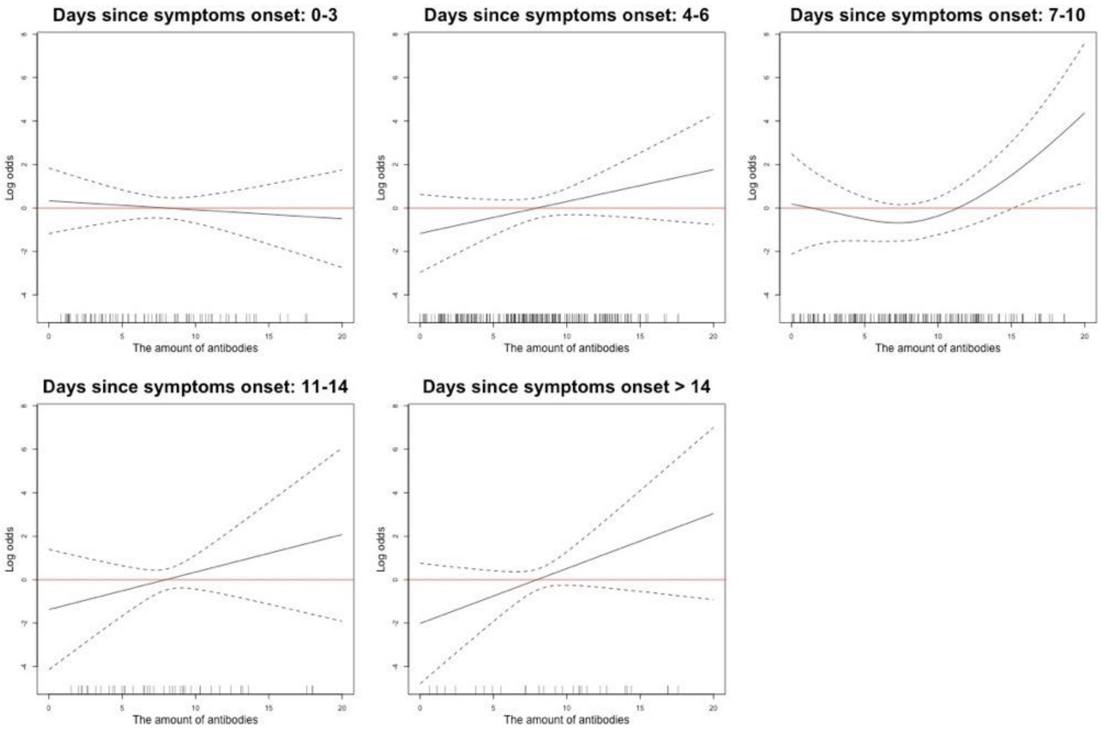

Figure S16. The patient's enrollment quarter group-specific dose-response association for the binary outcome of ventilation or death at day 14.

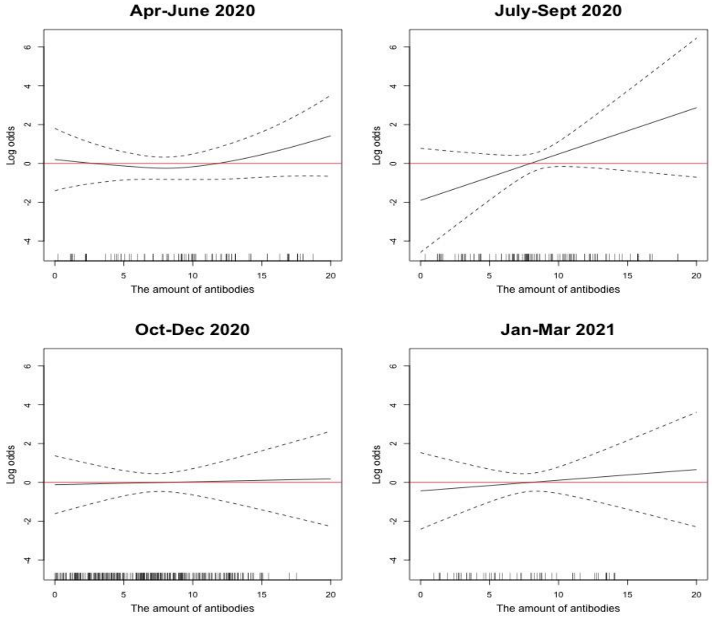

Figure S17. The concomitant medication antiplatelet group-specific dose-response association for the binary outcome of ventilation or death at day 14.

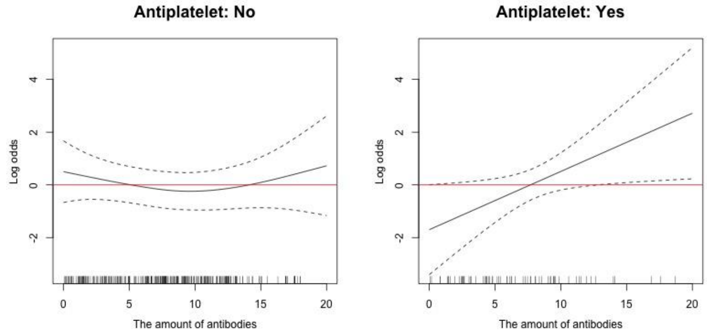

Figure S18. The concomitant medication anticoagulant group-specific dose-response association for the binary outcome of ventilation or death at day 14.

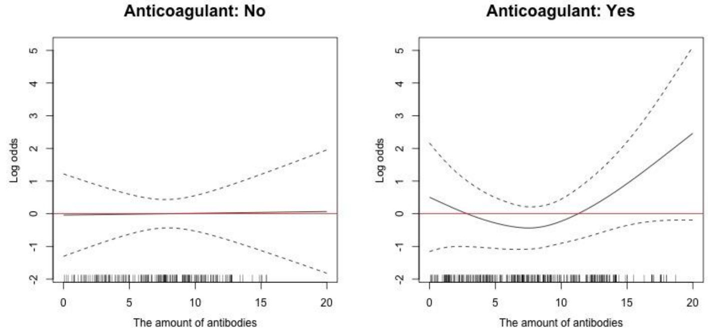

S2.3. Sensitivity analysis

We additionally conducted a sensitivity analysis to address potential selection bias due to excluding patients with missing antibody measures that may occur if the missingness is related to patient-level characteristics. Specifically, we applied an inverse propensity weighting (IPW) adjustment to the continuous dose analysis (n=576), using weights that are the inverse of the estimated propensity for missing antibody measures. The IPW-adjusted results are shown in **Figure S19**, with details on the weight computation using propensity score models provided in subsequent paragraphs titled "Propensity model estimation for sensitivity analysis."

By assigning weights based on the inverse of the propensity to the available cases (i.e., assigning higher weights to the observed cases with higher propensity of missingness), we aimed to create a weighted sample that reflects the characteristics of the entire population, including those with missing antibody measures.

As in the dose-response curves reported in Figure 2 of the main manuscript, the sensitivity analysis results in **Figure S19** also shows non-monotonic relationships between the dose of antibodies and the CCP treatment response in patients who require oxygen support at baseline (bottom row) while exhibiting a consistently positive dose-response in patients not requiring oxygen supplementation at baseline (top row).

As an additional sensitivity analysis, we conducted a weighted multivariable regression with IPW adjustment for interaction effects, shown in **Table S6**. The results are largely consistent (but generally with larger p-values) with those reported in **Table S3** of Supplementary Materials. Details of the construction of the propensity model for non-missingness of antibody measures are provided in the following paragraphs.

Propensity model estimation for sensitivity analysis.

In the propensity model building for non-missing antibody measure, we excluded the cases from the RCT in India (n=157) as the RCT in India did not collect any antibody information and solely contributed control-treated patients (see **Table S2**). Thus, a total of n=1074 CCP treated cases were considered in the propensity model building (see **Table S4** for a comparison between the missing and non-missing groups with respect to the observed outcomes). Among these CCP treated cases, we had n=576 non-missing and n=498 missing antibody measure cases, respectively.

Specifically, we modeled the propensity of "non-missing" antibody as a function of baseline variables (**Table S5**) using logistic regression. To address sporadic missing covariate data, we used the "mice" package in R to perform multiple imputation (MI) at the outset of the analysis. We generated 100 multiply-imputed complete datasets by including all available pre-treatment covariates (see **Table 1**) and employing predictive mean matching in the imputation model.

Since there was a relatively large number of baseline covariates that may cause concerns in terms of the estimation stability, we conducted variable selection by fitting elastic-net[14] regularized logistic regression models in developing this propensity model.

Specifically, to make this elastic-net based variable selection procedure more robust (by accounting for the uncertainties in the regularization parameter selection), we further bootstrapped each of the 100

[14] Zou, H. and Hastie, T. (2005). "Regularization and Variable Selection via the Elastic Net." Journal of the Royal Statistical Society. Series B (Statistical Methodology), 67(2), 301–320.

imputed datasets b=100 times, resulting in m×b = 10000 imputed and bootstrapped complete datasets. Then, for each of these m×b =10000 datasets, we fit an elastic-net regularized logistic regression model for the "non-missingness" of the antibody measure on all available baseline predictors (see the "Initial" model in **Table S5**, obtained from an unregularized logistic regression). For each dataset, we selected the sparsity regularization parameter via 10-fold cross-validation (using the built-in functionality of the R package "glmnet"[15,16]) and identified an active set of relevant predictors with non-zero regression coefficients. We report the percentage of times each predictor is selected across the fitted m×b =10000 elastic-net regularized models, in the second column of **Table S5**.

The baseline variables being selected by the elastic-net regularization more than 50% of the times out of the m×b =10000 fitted models were used as the predictors in the final propensity model. Using these selected baseline variables as predictors, we fit a logistic regression propensity model for "non-missing" antibody for each of the m=100 imputed datasets separately. To display the results, we subsequently pooled these regression coefficients across the 100 fits using Rubin's method; see **Table S5** for the "final" model pooled logistic regression coefficients.

Then we obtain a set of m=100 propensity score vectors, one for each of the m=100 imputed datasets: each propensity score vector has the propensity scores for non-missingness of the antibody measure for the n=1074 cases, specific to the corresponding imputed dataset. Utilizing these estimated propensity scores as the inverse of the weights, we applied IPW adjustment to the available cases used in the analysis (n=576). **Figure S19** presents the results of the continuous dose-outcome analyses under the IPW adjustment, and a set of weighted multivariable regression with IPW adjustment to explore moderating impacts of baseline factors are presented in **Table S6**.

[15] Friedman, J., Tibshirani, R., Hastie, T. (2010). "Regularization Paths for Generalized Linear Models via Coordinate Descent." Journal of Statistical Software, 33(1), 1–22.
[16] Tay, J.K., Narasimhan, B., Hastie, T. (2023). "Elastic Net Regularization Paths for All Generalized Linear Models." Journal of Statistical Software, 106(1), 1–31.

Figure S19. (IPW-adjusted sensitivity analysis) Dose-response curves for five clinical outcomes in CCP-treated patients, stratified by baseline oxygen support status (WHO score of 4, no oxygen support required, in the top row; WHO score of 5 or 6, oxygen support required, in the bottom row), adjusting for the expanded covariate and concomitant medication sets. The x-axis represents antibody dose measured on OrthoV, and the y-axis represents the log odds of an unfavorable outcome, with lower values indicating a more favorable clinical outcome.

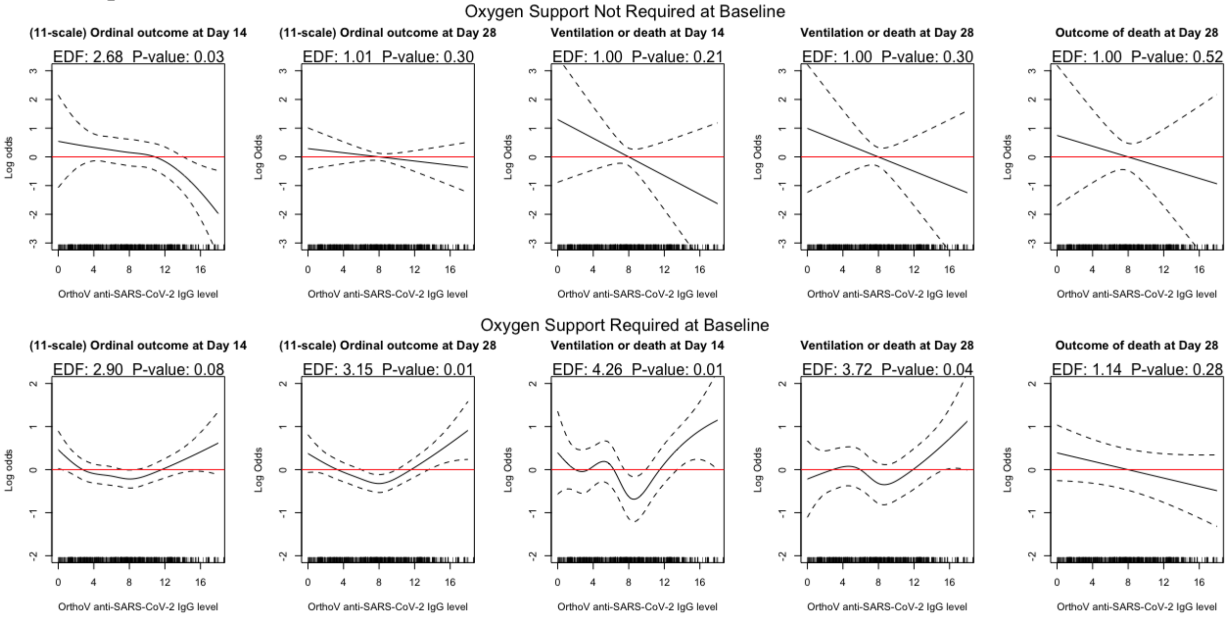

Table S4. Comparison of the outcomes between the missing and non-missing antibody measure groups among the n=1074 CCP treated patients (excluding data from the RCT in India) that were used to build a propensity model for non-missing antibody measure. For the (11-scale) ordinal outcomes, Wilcoxon test was conducted for the difference between the two groups. For the binary outcomes, proportion test was conducted for the difference between the two groups.

	Missing antibody measure group	Non-missing antibody measure group	P-value associated with the difference in the outcome
n = 1074	n = 498	n = 576	
WHO 11-scale at Day 14			0.1588
WHO= 0	65 (13.1%)	61 (10.6%)	
WHO= 1	62 (12.4%)	53 (9.2%)	
WHO= 2	174 (34.9%)	200 (34.7%)	
WHO= 3	35 (7.0%)	86 (14.9%)	
WHO= 4	14 (2.8%)	24 (4.2%)	
WHO= 5	35 (7.0%)	49 (8.5%)	
WHO= 6	30 (6.0%)	18 (3.1%)	
WHO= 7	12 (2.4%)	19 (3.3%)	
WHO= 8	20 (4.0%)	18 (3.1%)	
WHO= 9	12 (2.4%)	11 (1.9%)	
WHO= 10	31 (6.2%)	32 (5.6%)	
NA	8 (1.6%)	5 (0.9%)	
WHO 11-scale at Day 28			0.0109
WHO= 0	130 (26.1%)	106 (18.4%)	
WHO= 1	56 (11.2%)	53 (9.2%)	
WHO= 2	164 (32.9%)	220 (38.2%)	
WHO= 3	32 (6.4%)	73 (12.7%)	
WHO= 4	8 (1.6%)	17 (3.0%)	
WHO= 5	15 (3.0%)	17 (3.0%)	
WHO= 6	6 (1.2%)	5 (0.9%)	
WHO= 7	7 (1.4%)	11 (1.9%)	
WHO= 8	7 (1.4%)	5 (0.9%)	
WHO= 9	13 (2.6%)	3 (0.5%)	
WHO= 10	52 (10.4%)	59 (10.2%)	
NA	8 (1.6%)	7 (1.2%)	
Binary outcome of ventilation or worse at day 14 (%)	75 (15.1%)	80 (13.9%)	0.6472
Binary outcome of ventilation or worse at day 28 (%)	79 (15.9%)	78 (13.5%)	0.3234
Binary outcome of death at day 14 (%)	31 (6.2%)	32 (5.6%)	0.7374
Binary outcome of death at day 28 (%)	52 (10.4%)	59 (10.2%)	0.9951

Table S5. The estimated propensity models for antibody measure non-missingness. Predictors for the 'Final propensity model' were selected based on a 50% cut-off from (100 imputation x 100 bootstrap =) 10,000 datasets, where we fit elastic-net regularized logistic regression to select predictors from the "Initial propensity model" for each dataset.

		**Initial propensity model** (pooled by Rubin's method across the 100 imputed datasets)				**Final propensity model** (pooled by Rubin's method across the 100 imputed datasets)			
Coefficient	P(select)	Estimate	St.Err.	z	p-value	Estimate	St.Err.	z	p-value
(Intercept)		-2.66	1.01	-2.63	0.008	-1.3	0.63	-2.1	0.009
RCT: UPenn	**100%**	4.18	0.80	5.22	< 10^-3	4.2	0.78	5.33	< 10^-3
RCT: Spain	**100%**	4.79	0.59	8.07	< 10^-3	4.75	0.57	8.32	< 10^-3
RCT: UCSF	**100%**	17.7	952	0.01	0.985	17.9	951	0.02	0.985
RCT: Belgium	**84%**	0.46	0.25	1.8	0.072	0.28	0.22	1.24	0.216
RCT: Brazil	**100%**	3.33	0.86	3.87	< 10^-3	3.07	0.83	3.69	< 10^-3
RCT: Netherlands	**100%**	19.5	631	0.03	0.975	19.3	636	0.03	0.976
Age	**56%**	0.0119	0.006	1.77	0.077	0.0099	0.0062	1.59	0.112
Sex Female	44%	0.15	0.18	0.86	0.388				
Blood type A	47%	0.09	0.18	0.50	0.617	0.06	0.17	0.33	0.737
Blood type B	24%	0.15	0.26	0.58	0.56	0.15	0.26	0.59	0.553
Blood type AB	**51%**	-0.33	0.44	-0.75	0.453	-0.3	0.44	-0.68	0.496
Weight	37%	0.0054	0.004	1.25	0.211				
Systolic BP	41%	-0.001	0.004	-0.19	0.844				
Asthma	**70%**	-0.42	0.28	-1.51	0.132	-0.37	0.27	-1.34	0.180
Diabetes	37%	0.08	0.18	0.45	0.651				
Pulmonary disease	30%	0.11	0.30	0.38	0.702				
Cardiovascular disease	**52%**	0.01	0.19	0.07	0.942	0.03	0.18	0.21	0.833
Days onset 4-6	40%	-0.01	0.27	-0.06	0.951	-0.02	0.26	-0.10	0.914
Days onset 7-10	24%	-0.04	0.27	-0.16	0.866	-0.06	0.26	-0.23	0.818
Days onset 11-14	**72%**	-0.37	0.36	-1.03	0.304	-0.42	0.35	-1.2	0.231
Days onset >14	41%	0.03	0.41	0.08	0.933	-0.03	0.40	-0.09	0.922
July-Sept 2020	**99%**	1.72	0.39	4.34	< 10^-3	1.7	0.35	4.74	< 10^-3
Oct-Dec 2020	**100%**	2.42	0.38	6.23	< 10^-3	2.4	0.34	7.07	< 10^-3
Jan-Mar 2021	**70%**	0.53	0.43	1.24	0.217	0.55	0.38	1.44	0.151
Antiplatelet	**81%**	-0.36	0.21	-1.68	0.092	-0.35	0.21	-1.68	0.093
Anticoagulant	**97%**	0.35	0.19	1.89	0.059	0.46	0.18	2.54	0.011
Renal replacement	**78%**	0.69	0.51	1.37	0.172	0.66	0.50	1.33	0.185
Serostatus (+)	**55%**	0.08	0.28	0.28	0.778	0.06	0.28	0.22	0.826
Hydroxychloroquine	17%	0.50	0.58	0.86	0.39				
Antibacterial	44%	0.18	0.18	1.01	0.314				
Antiviral	45%	0.15	0.51	0.29	0.769				
Remdesivir	41%	0.31	0.22	1.36	0.173				
Anti-inflammatory	**73%**	-0.43	0.25	-1.73	0.083	-0.31	0.24	-1.28	0.202
Steroids	**98%**	-0.73	0.22	-3.24	0.001	-0.69	0.22	-3.12	0.001
Antithrombotic	**87%**	-0.22	0.27	-0.81	0.413	-0.26	0.26	-0.98	0.325
WHO = 5	36%	-0.44	0.37	-1.19	0.236	-0.38	0.37	-1.03	0.302
WHO = 6	**60%**	-0.60	0.42	-1.44	0.151	-0.46	0.41	-1.12	0.263

Table S6. (IPW-adjusted sensitivity analysis) Results of interaction analyses for CCP dose and baseline factors on each outcome. We investigated the potential (possibly nonlinear) dose-by-baseline covariate interactions, while adjusting for the expanded covariate and concomitant medication sets, based on generalized additive cumulative logistic or logistic regression models. Separately for each baseline factor, we assessed the baseline covariate's moderating impact on dose-outcome relationships by examining the significance of the factor’s level-specific dose-response curves, capturing distinct associations beyond the factor’s reference level dose-response curve. We provide median values of effective degrees of freedom (EDF) and P-values across 100 imputed datasets to quantify nonlinearity and the strength of the association beyond the reference level's dose-response.

	11-scale ordinal at day 14		11-scale ordinal at day 28		Ventilation or worse at day 14		Ventilation or worse at day 28		Death at day 28	
	EDF	P-value	EDF	P-value	EDF	P-value	EDF	P-value	EDF	P-value
RCT: NYC (ref)										
RCT: UPenn	1.00	0.344	1.00	0.192	1.83	0.894	1	**0.099**	1	0.112
RCT: Spain	1.00	0.606	1.00	0.718	1.00	0.881	1	0.347	1.00	0.703
RCT: UCSF	1.46	0.221	1	0.787	1.98	1	1.98	1	1.98	1
RCT: Belgium	4.74	**0**	4.76	**0**	3.88	**0.025**	2.33	**0.001**	2.08	**0.004**
RCT: Brazil	1	**0.001**	1	**0.030**	3.63	1	2.94	1	1.02	0.584
RCT: Netherland	2.67	**0.008**	2.04	0.173	1.63	0.216	1.01	0.041	1.70	0.183
Age <=50 (ref)										
Age (50,65]	1.25	0.854	1.00	0.608	2.10	0.250	1	0.928	2.89	**0.013**
Age > 65	4.43	**0**	2.74	**0.02**	1.00	**0.047**	2.85	**0.025**	1	**0.084**
Sex Female	1.00	0.152	3.90	**0.016**	4.26	0.102	4.49	**0.002**	4.58	**0**
Blood type O (ref)										
Blood type A	1.80	**0.037**	1.00	**0.014**	1	**0**	1	**0.01**	1	0.108
Blood type B	1.71	0.452	1.97	0.250	1.65	**0**	2.32	**0.089**	4.45	**0.001**
Blood type AB	1.00	0.456	3.97	**0.013**	3.75	**0.051**	2.22	0.48	2.23	0.769
Systolic BP (>128)	1.77	0.516	1.00	**0.064**	2.01	**0**	1	**0.05**	2.68	0.19
Weight (>90kg)	3.95	**0**	4.17	**0**	4.54	**0**	3.68	**0.007**	4.25	**0.001**
Asthma	2.49	**0.016**	1.56	**0.060**	2.46	**0.075**	2.58	0.144	1.65	0.594
Diabetes	3.49	0.124	1.00	0.221	4.33	**0**	4.19	**0.021**	3.96	**0.072**
Pulmonary disease	1.00	**0.032**	1.00	**0.046**	1.39	0.304	1	**0.097**	1	0.274
Cardiovascular disease	1.00	**0.002**	2.22	**0.020**	3.03	**0.033**	1	0.744	2.95	**0.048**
Days onset 0-3 (ref)										
Days onset 4-6	3.62	0.134	1.00	**0.089**	4.43	**0.053**	3.79	**0.073**	2.33	0.13
Days onset 7-10	3.40	**0**	3.11	**0**	3.27	**0.001**	2.82	**0.004**	3.19	**0.015**
Days onset 11-14	2.89	**0.001**	3.46	**0.003**	3.09	1	3.40	1	1.93	1
Days onset >14	2.53	**0.034**	2.00	**0.006**	3.78	**0.062**	3.78	**0.075**	3.80	**0.045**
Apr-June 2020 (ref)										
July-Sept 2020	3.79	**0.01**	3.19	**0.015**	1	**0.059**	1.00	**0**	1	**0**
Oct-Dec 2020	2.99	0.259	3.41	**0.021**	1	0.620	1.00	**0.036**	1	0.318
Jan-Mar 2021	1.00	**0.054**	1.00	0.484	4.68	**0.005**	3.57	**0.022**	3.7	**0.014**
Antiplatelet	3.93	**0**	4.27	**0**	4.16	**0.001**	3.77	**0.01**	4.51	**0**
Anticoagulant	1.00	0.441	1.74	0.534	2.80	**0.091**	3.06	0.188	1.97	0.611
Renal therapy	1.00	0.567	1.00	0.558	1	0.802	1	0.8	1	0.778
Serostatus (+)	2.15	0.227	3.51	**0.050**	1	0.383	1	0.756	1	0.502
HCQ sulfate	2.33	0.194	1.23	0.440	1	0.174	1	1	1	1
Antibacterial	4.53	**0**	3.12	**0.012**	4.69	**0.001**	3.04	0.14	3.00	0.276
Antiviral	1.00	**0**	1.00	**0.018**	1	0.522	1	0.183	1	0.637
Remdesivir	1.00	0.413	3.74	**0.065**	1.08	0.350	1.00	0.33	1	0.379
Anti-inflammatory	1.00	0.289	1.05	0.902	1	**0.051**	2.08	0.289	1.00	0.207
Steroids	1.00	0.129	1.56	**0.074**	1	0.178	1	0.882	1	0.701
Antithrombosis	4.27	0.100	3.56	**0.088**	1	0.677	2.63	0.357	1	0.830
WHO = 4 (ref)										
WHO = 5	2.60	**0.003**	2.91	**0.001**	4.26	**0.001**	3.94	**0.013**	2.60	0.362
WHO = 6	1.00	0.117	1.00	0.935	1.00	0.745	1	0.495	1.21	0.558

Section S3. Antibody group analysis

S3.1. Main dose group analysis

In the dose group analysis, we compared the outcomes between the SARS-CoV-2 IgG levels (higher dose: ≥8 S/Co, and lower dose: <8 S/Co, where the S/Co value of 8 corresponds to the observed mean of the OrthoV measure) and the control group. To evaluate the dose groups' CCP efficacy (vs. control), we used Bayesian multivariable regression models (logistic regression for binary outcomes and cumulative logit proportion odds regression for ordinal outcomes) with the dose group (with 3 levels: control, lower and higher dose) as the main regressor, while adjusting for the **expanded covariate** and **concomitant medication** sets.

For the regression coefficients associated with the group indicators and the covariates, we used weakly informative Gaussian priors with mean zero and standard deviation of 2.5, which conservatively estimate the regression parameters to reduce type I error rates, mitigating the need for post-hoc corrections for multiple comparisons.

To address sporadic missing covariate data, we used the "mice" package in R to perform multiple imputation at the outset of the analysis as discussed in Section S1. Of note, we imputed only the missing baseline covariates, not the outcomes nor the dose information. We generated 100 multiply-imputed complete datasets by including all available pre-treatment covariates (see **Table 1**) and employing predictive mean matching in the imputation model. We combined the analysis results from the 100 imputed datasets by aggregating the posterior draws of CCP efficacy ORs into a single comprehensive posterior distribution[17]. Figure 3 in the main manuscript shows the dose group-specific CCP efficacy ORs, stratified by baseline oxygen support status, adjusted for expanded covariates and concomitant medications.

The unstratified analysis results, presented in **Figure S20**, not stratified by baseline oxygen supplementation status, displayed also unclear dose-response patterns, except for a superior day 28 mortality benefit of higher-dose CCP (/control) (OR =0.69, CrI: [0.38, 1.19], Pr(OR<1) =0.91) compared to lower-dose CCP (/control) (OR =1.04, CrI: [0.65, 1.63], Pr(OR<1) =0.44).

Mirroring the analyses conducted with stratification by baseline oxygen support status, similar analyses were conducted by stratifying by days since symptom onset to treatment initiation (≤3 vs. >3 days). The resulting dose group-specific CCP efficacy ORs (and 80% and 95% credible intervals), stratified by days since symptom onset, are displayed in **Figure S21**. In patients with ≤3 days since symptom onset (who are likely in an earlier disease stage), a higher CCP dose showed stronger effectiveness signals that a lower CCP dose, which is consistent with the findings stratified by the baseline oxygen support status presented in the main manuscript.

In **Table S7**, we report a comparison of raw outcomes between the groups (control, lower and higher dose). The primary outcome event of receiving mechanical ventilatory support or death at day 14 post-treatment occurred in 15.7% (179 of 1138 patients) in the control group, 14.5% (44 of 304 patients) in the lower dose group, and 13.2% (36 of 272 patients) in the higher dose group. In **Table S7**, we also report the unadjusted CCP efficacy ORs comparing the two dose groups with the control group.

---

[17] Zhou, X. and Reiter, J.P. (2010). "A Note on Bayesian Inference After Multiple Imputation." The American Statistician 64, 159-163.

To identify baseline covariates that possibly had influenced the antibody dose group assignments, we estimated 3 propensity models corresponding to the 3 possible pairs of the dose group assignments (Higher vs. Lower, Higher vs. Control, and Lower vs. Control). We utilized Bayesian logistic regression for the dose group assignment with hierarchical shrinkage priors (regularized horseshoe[18], using the default hyperparameters implemented in the R package “rstanarm” version 2.21.1) to prevent overfitting. The regression coefficients of the estimated propensity models are summarized in **Figures S22-S24**. Besides the RCT IDs that showed strong association with the (higher vs. lower) dose group assignment, there were moderate degrees of association between the propensity of the dose group assignments and anti-inflammatory, anti-viral, hydroxychloroquine or antithrombotic agent use, patient enrollment quarters, patient blood types and the baseline WHO score, all of which were adjusted for or stratified in our analyses.

[18] Piironen, J. and Vehtari, A. (2017). “Sparsity information and regularization in the horseshoe and other shrinkage priors.” Electronic Journal of Statistics, Vol. 11 (2017) 5018–5051.

Table S7. Raw outcomes (count and within-group percentage) and unadjusted CCP efficacy ORs (and 95% credible intervals) for 5 outcomes, in the CCP (lower or higher) dose groups vs. control group.

	Control	Lower dose (< 8)	Higher dose (≥ 8)
	n=1138	n=304	n=272
**1. WHO 11-scale ordinal outcome at day 14 post-treatment**			
WHO= 0	n= 120 (10.5%)	n= 27 (8.9%)	n= 34 (12.5%)
WHO= 1	n= 151 (13.3%)	n= 26 (8.6%)	n= 27 (9.9%)
WHO= 2	n= 370 (32.5%)	n= 105 (34.5%)	n= 95 (34.9%)
WHO= 3	n= 141 (12.4%)	n= 47 (15.5%)	n= 39 (14.3%)
WHO= 4	n= 45 (4.0%)	n= 13 (4.3%)	n= 11 (4.0%)
WHO= 5	n= 89 (7.8%)	n= 23 (7.6%)	n= 26 (9.6%)
WHO= 6	n= 30 (2.6%)	n= 14 (4.6%)	n= 4 (1.5%)
WHO= 7	n= 25 (2.2%)	n= 9 (3.0%)	n= 10 (3.7%)
WHO= 8	n= 24 (2.1%)	n= 7 (2.3%)	n= 11 (4.0%)
WHO= 9	n= 33 (2.9%)	n= 6 (2.0%)	n= 5 (1.8%)
WHO= 10	n= 97 (8.5%)	n= 22 (7.2%)	n= 10 (3.7%)
NA	n= 13 (1.1%)	n= 5 (1.6%)	n= 0 (0.0%)
Unadjusted CCP efficacy Cumulative OR (vs. control) [95% CI]		1.16 [0.94, 1.46]	0.94 [0.74, 1.18]
**2. WHO 11-scale ordinal outcome at day 28 post-treatment**			
WHO= 0	n= 246 (21.6%)	n= 58 (19.1%)	n= 48 (17.6%)
WHO= 1	n= 195 (17.1%)	n= 22 (7.2%)	n= 31 (11.4%)
WHO= 2	n= 330 (29.0%)	n= 113 (37.2%)	n= 107 (39.3%)
WHO= 3	n= 95 (8.3%)	n= 36 (11.8%)	n= 37 (13.6%)
WHO= 4	n= 26 (2.3%)	n= 10 (3.3%)	n= 7 (2.6%)
WHO= 5	n= 28 (2.5%)	n= 12 (3.9%)	n= 5 (1.8%)
WHO= 6	n= 14 (1.2%)	n= 3 (1.0%)	n= 2 (0.7%)
WHO= 7	n= 15 (1.3%)	n= 4 (1.3%)	n= 7 (2.6%)
WHO= 8	n= 9 (0.8%)	n= 1 (0.3%)	n= 4 (1.5%)
WHO= 9	n= 11 (1.0%)	n= 1 (0.3%)	n= 2 (0.7%)
WHO= 10	n= 153 (13.4%)	n= 39 (12.8%)	n= 20 (7.4%)
NA	n= 16 (1.4%)	n= 5 (1.6%)	n= 2 (0.7%)
Unadjusted CCP efficacy Cumulative OR (vs. control) [95% CI]		1.30 [1.05, 1.62]	1.12 [0.89, 1.41]
**3. Binary outcome of ventilation or death at day 14 post-treatment**			
0 (WHO < 7)	n= 946 (83.1%)	n= 255 (83.9%)	n= 236 (86.85%)
1 (WHO ≥ 7)	n= 179 (15.7%)	n= 44 (14.5%)	n= 36 (13.2%)
NA	n= 13 (1.1%)	n= 5 (1.6%)	n= 0 (0.0%)
Unadjusted CCP efficacy OR (vs. control) [95% CI]		0.91 [0.63, 1.30]	0.80 [0.55, 1.16]
**4. Binary outcome of ventilation or death at day 28 post-treatment**			
0 (WHO < 7)	n= 934 (82.1%)	n= 254 (83.6%)	n= 237 (87.1%)
1 (WHO ≥ 7)	n= 188 (16.5%)	n= 45 (14.8%)	n= 33 (12.1%)

NA	n= 16 (1.4%)	n= 5 (1.6%)	n= 2 (0.7%)
Unadjusted CCP efficacy OR (vs. control) [95% CI]		0.87 [0.60, 1.26]	0.68 [0.45, 1.01]
**5. Binary outcome of death at day 28 post-treatment**			
0 (alive)	n= 969 (85.1%)	n= 260 (85.5%)	n= 250 (91.9%)
1 (death)	n= 153 (13.4%)	n= 39 (12.8%)	n= 20 (7.4%)
NA	n= 16 (1.4%)	n= 5 (1.6%)	n= 2 (0.7%)
Unadjusted CCP efficacy OR (vs. control) (95% CI)		0.95 [0.64, 1.39]	0.50 [0.29, 0.81]

Figure S20. The CCP efficacy odds ratios (and 80% and 95% credible intervals) of the antibody dose groups [the antibody dose range divided into two categories: Lower dose (<8 S/Co) and Higher dose (≥8 S/Co)] in reference to the control group for each of the 5 outcomes, **not stratified by the baseline oxygen supplementation status**, adjusted for the expanded and concomitant medication sets; the odds ratios < 1 indicate a greater CCP efficacy vs. control.

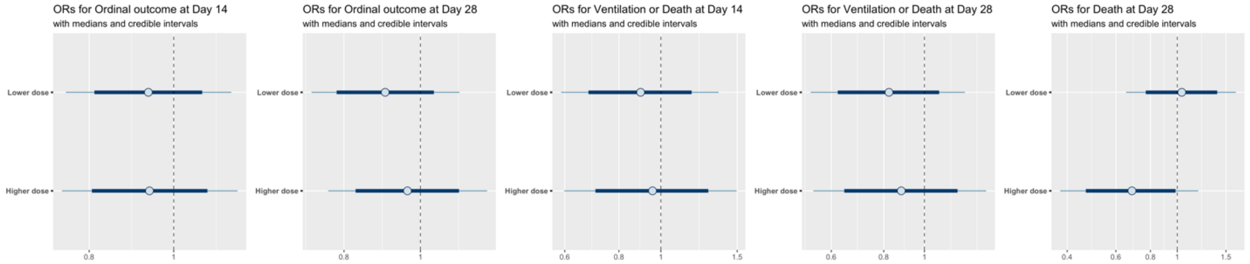

Figure S21. The CCP efficacy odds ratios (and 80% and 95% credible intervals) of the antibody dose groups [the antibody dose range divided into two categories: Lower dose (<8 S/Co) and Higher dose (≥8 S/Co)] in reference to the control group for each of the 5 outcomes, stratified by the **time since symptom onset (≤ 3 days vs. > 3 days)**, adjusted for the expanded and concomitant medication sets; the odds ratios < 1 indicate a greater CCP efficacy vs. control.

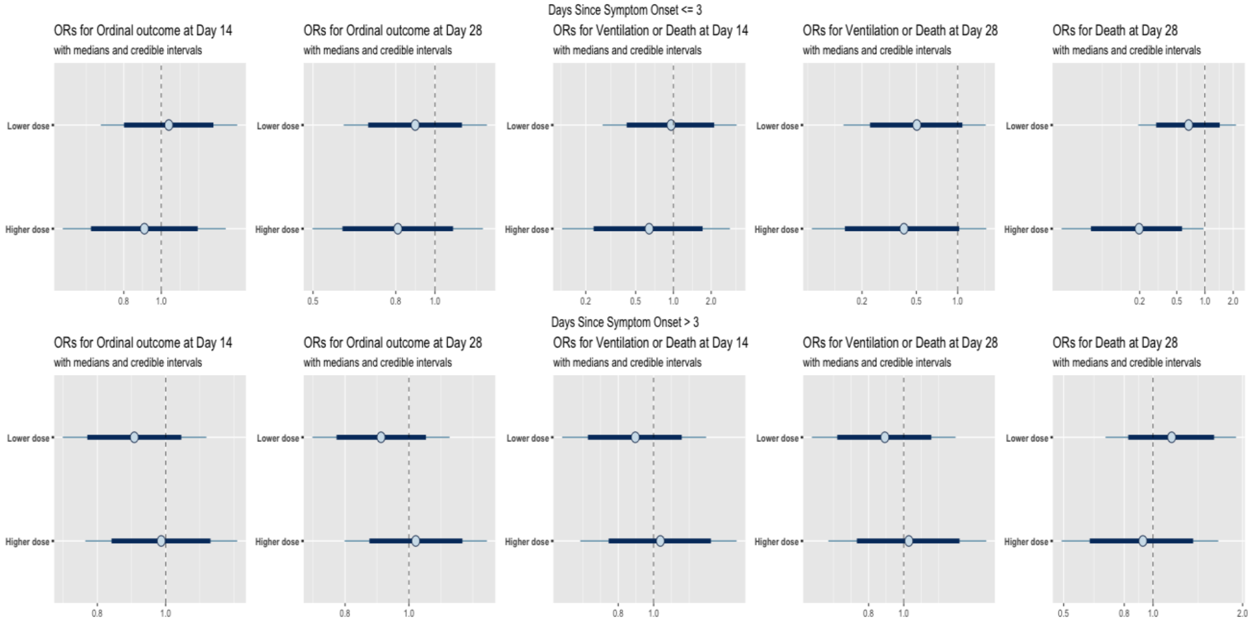

Figure S22. Propensity (higher vs. lower dose groups) logistic regression model coefficients.

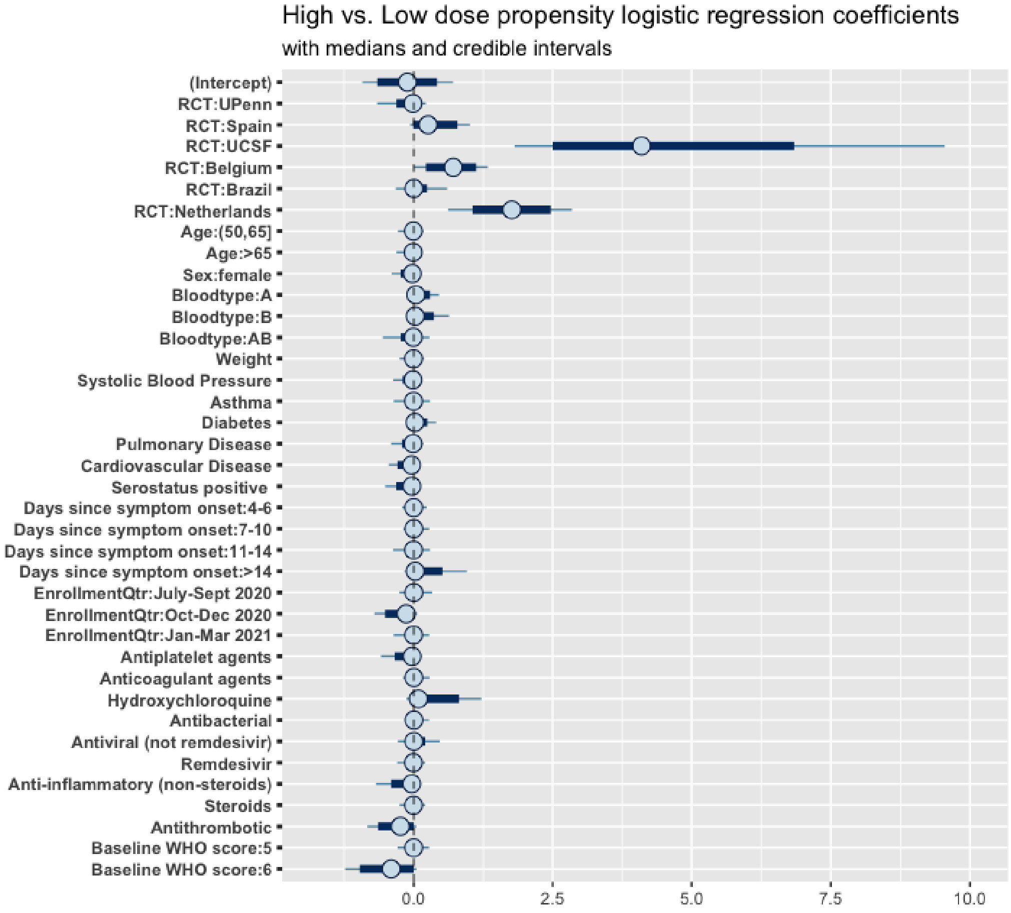

Figure S23. Propensity (higher dose vs. control groups) logistic regression model coefficients.

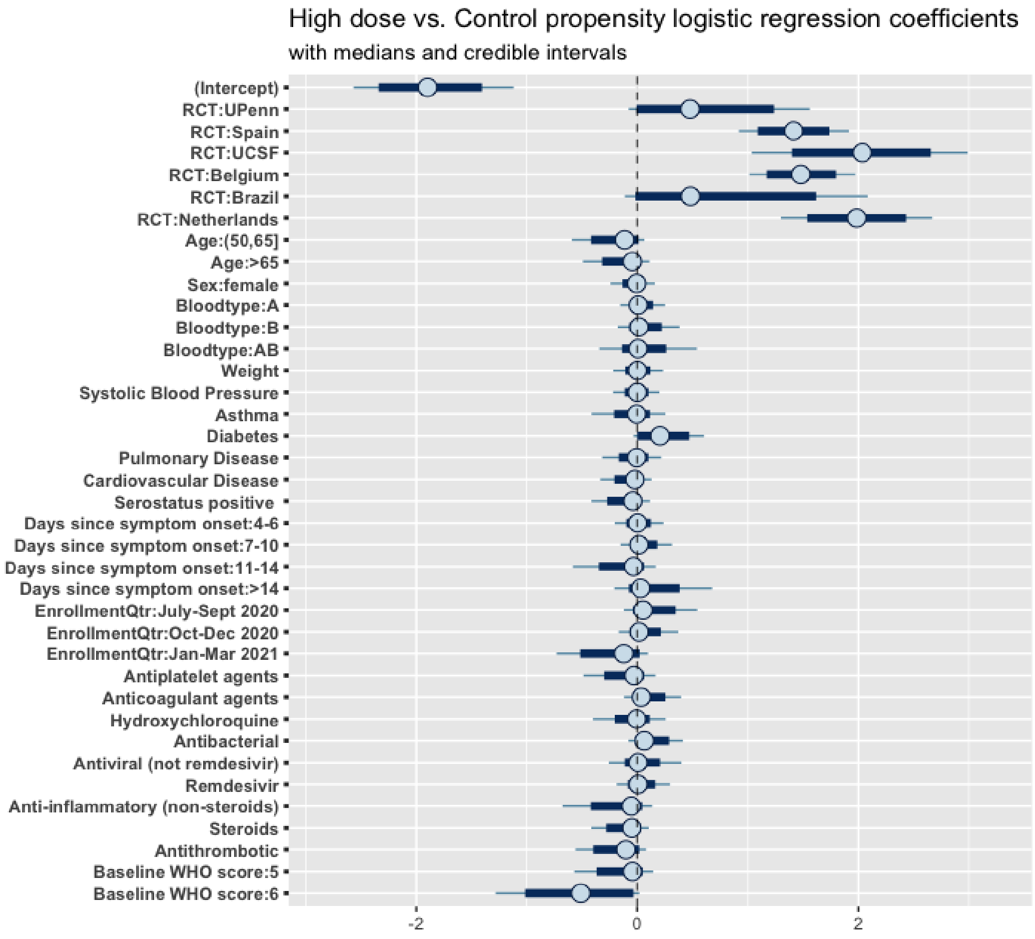

Figure S24. Propensity (lower dose vs. control groups) logistic regression model coefficients.

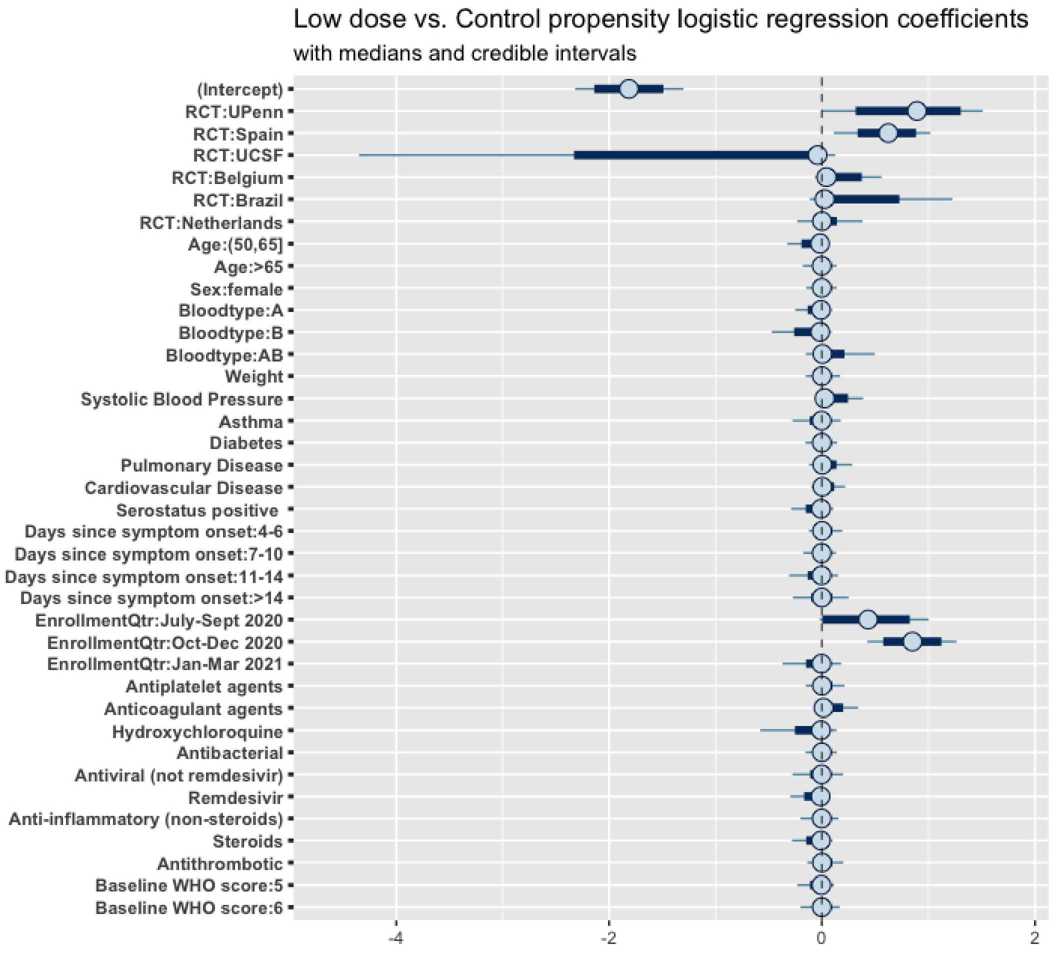

S3.2. Dose group-by-covariates interaction analysis

In **Table S8**, we present subgroup-specific CCP efficacy ORs for the primary outcome of ventilation or death at day 14 post-treatment, adjusted for the expanded covariate set, where subgroups are defined by baseline factors, including participating RCT, patient enrollment quarter, baseline WHO score, days since symptom onset, age, sex, blood type, and history of cardiovascular disease, diabetes, or pulmonary disease. As an exploratory analysis, we also categorized the study population into two TBI subgroups: low CCP benefit (TBI <0.35) and High CCP benefit (TBI ≥ 0.35) (see Tables S1 and Table 1) groups, and assessed the dose group-specific CCP efficacy ORs within these TBI subgroups, adjusted for study enrollment quarters, RCT IDs and the concomitant medication set.

Additionally, we conducted multivariable regression to explore potential CCP effect variations by baseline factors (comorbidity, concomitant medications, disease severity) on the binary outcome of ventilation or death at day 14. As there were 3 groups (including the control group), we considered three pairwise group comparisons: 1) Higher dose vs. control; 2) Lower dose vs. control; 3) Higher dose vs. Lower dose, when we investigated the CCP effect variations. We used Bayesian logistic regression that included main effects for dose group and baseline factors, as well as interaction effects between them. All relevant baseline factors were included in a single regression model to prevent spurious findings due to multiplicity, and to prevent overfitting, we used hierarchical Bayesian shrinkage priors (regularized horseshoe[19]). This approach allowed us to examine the effects of multiple covariates-by-dose group interactions, while avoiding the challenges associated the need for adjusting for multiple comparison in conducting subgroup analyses.

In **Figures S25-S27**, we present the estimated (exponentiated) regression coefficients (along with their 80% and 95% credible intervals) of the dose group-by-baseline covariates interaction effects. These exponentiated regression coefficients represent each baseline factor level's multiplicative impact on the CCP efficacy odds ratio, compared to its reference level. It appears that in **Figure S25**, the efficacy of lower dose (vs. control) was higher in a particular quarter (July-September 2020) compared to the other quarters.

[19] Piironen, J. and Vehtari, A. (2017). "Sparsity information and regularization in the horseshoe and other shrinkage priors." Electronic Journal of Statistics, Vol. 11 (2017) 5018–5051.

Table S8. CCP dose group-specific efficacy ORs compared to control (along with 95% credible intervals), with respect to the primary binary outcome of ventilation or death at day 14, adjusted for the expanded covariate set.

	Dose group		[marginal]
	Lower dose (<8 S/Co) (n=304)	Higher dose (≥8 S/Co) (n=272)	
RCT			
- NYC (n=614)	0.80 [0.37, 1.60]	1.08 [0.36, 2.93]	0.85 [0.57, 1.26]
- UPenn (n=78)	0.12 [0.01, 1.06]	0.11 [0.00, 2.68]	0.08 [0.01, 0.66]
- Spain (n=346)	0.43 [0.15, 1.09]	0.80 [0.31, 1.88]	0.58 [0.27, 1.22]
- UCSF (n=34)	- (no cases)	1.87 [0.06, 70.55]	1.91 [0.06, 72.78]
- Belgium (n=314)	1.97 [0.78, 5.04]	0.77 [0.27, 2.05]	1.14 [0.59, 2.29]
- Brazil (n=32)	11.03 [0.66, 205.5]	0.20 [0.00, 7.31]	2.07 [0.16, 31.61]
- Netherlands (n=72)	0.61 [0.02, 14.01]	5.86 [1.04, 34.96]	4.33 [0.77, 25.99]
Enrollment quarter			
- Apr-June 2020 (n=441)	0.32 [0.05, 1.45]	0.80 [0.27, 2.24]	0.71 [0.44, 1.14]
- July-Sept 2020 (n=334)	0.12 [0.02, 0.45]	0.55 [0.18, 1.55]	0.46 [0.24, 0.90]
- Oct-Dec 2020 (n=708)	1.33 [0.77, 2.32]	1.05 [0.48, 2.15]	1.02 [0.65, 1.60]
- Jan-Mar 2021 (n=231)	1.33 [0.35, 4.48]	1.48 [0.33, 6.07]	1.54 [0.77, 3.13]
Baseline WHO			
- 4 (n=312)	0.98 [0.10, 7.15]	0.28 [0.02, 2.14]	0.37 [0.14, 0.86]
- 5 (n=1109)	0.88 [0.49, 1.53]	0.98 [0.56, 1.71]	1.03 [0.73, 1.46]
- 6 (n=293)	0.80 [0.39, 1.65]	0.59 [0.17, 1.74]	0.89 [0.56, 1.41]
Days since symptom onset			
- 0-3 (n=218)	0.70 [0.19, 2.28]	0.60 [0.14, 2.37]	0.79 [0.37, 1.70]
- 4-6 (n=628)	1.14 [0.61, 2.10]	0.69 [0.31, 1.40]	0.85 [0.56, 1.27]
- 7-10 (n=592)	0.75 [0.32, 1.71]	0.91 [0.38, 2.00]	0.93 [0.58, 1.48]
- 11-14 (n=176)	0.17 [0.01, 1.50]	0.74 [0.05, 7.01]	0.94 [0.35, 2.42]
- >14 (n=85)	0.64 [0.04, 6.54]	3.04 [0.42, 21.97]	0.70 [0.21, 2.33]
Age			
- Age ≤ 50 (n=428)	0.26 [0.02, 1.59]	0.97 [0.20, 3.97]	0.93 [0.45, 1.95]
- 50 < Age ≤ 65 (n=606)	0.66 [0.26, 1.53]	1.75 [0.77, 3.91]	0.84 [0.52, 1.33]
- Age > 65 (n=680)	1.14 [0.66, 1.95]	0.60 [0.30, 1.16]	0.94 [0.66, 1.36]
Blood type			
- O (n=788)	0.84 [0.44, 1.56]	1.43 [0.72, 2.80]	1.02 [0.69, 1.51]
- A (n=589)	0.58 [0.28, 1.17]	0.52 [0.23, 1.11]	0.50 [0.32, 0.79]
- B (n=257)	4.39 [1.02, 19.62]	0.52 [0.07, 2.99]	2.05 [1.05, 4.05]
- AB (n=59)	1.21 [0.10, 14.16]	0.09 [0.00, 1.87]	0.28 [0.05, 1.58]
Cardio			
- No (n=938)	1.50 [0.77, 2.87]	1.10 [0.51, 2.31]	1.18 [0.82, 1.68]
- Yes (n=772)	0.55 [0.31, 0.96]	0.67 [0.36, 1.25]	0.64 [0.45, 0.94]
Diabetes			
- No (n=1159)	1.34 [0.80, 2.25]	1.10 [0.59, 1.96]	1.24 [0.90, 1.74]
- Yes (n=555)	0.39 [0.17, 0.81]	0.45 [0.20, 0.94]	0.50 [0.32, 0.75]
Pulmonary			
- No (n=1489)	0.92 [0.58, 1.42]	0.97 [0.59, 1.58]	0.91 [0.70, 1.21]
- Yes (n=220)	0.59 [0.18, 1.85]	0.46 [0.10, 1.82]	0.69 [0.31, 1.41]
Sex			
- Male (n=1093)	0.87 [0.50, 1.43]	0.93 [0.52, 1.59]	0.90 [0.65, 1.23]
- Female (n=621)	0.76 [0.35, 1.60]	0.70 [0.27, 1.64]	0.85 [0.52, 1.39]
TBI			
- Low (<0.35) (n=1034)	1.26 [0.77, 2.02]	1.19 [0.69, 2.01]	1.24 [0.83, 1.84]
- High (≥0.35) (n=645)	0.60 [0.28, 1.21]	0.34 [0.13, 0.78]	0.47 [0.25, 0.87]
[marginal]	0.94 [0.64, 1.36]	0.83 [0.53, 1.26]	

Figure S25. The estimated (exponentiated) regression coefficients (along with 80% and 95% credible intervals) of the dose group-by-baseline covariates interaction effects, each representing the corresponding baseline factor level's multiplicative impact on the efficacy OR (higher dose vs. control), compared to its reference level.

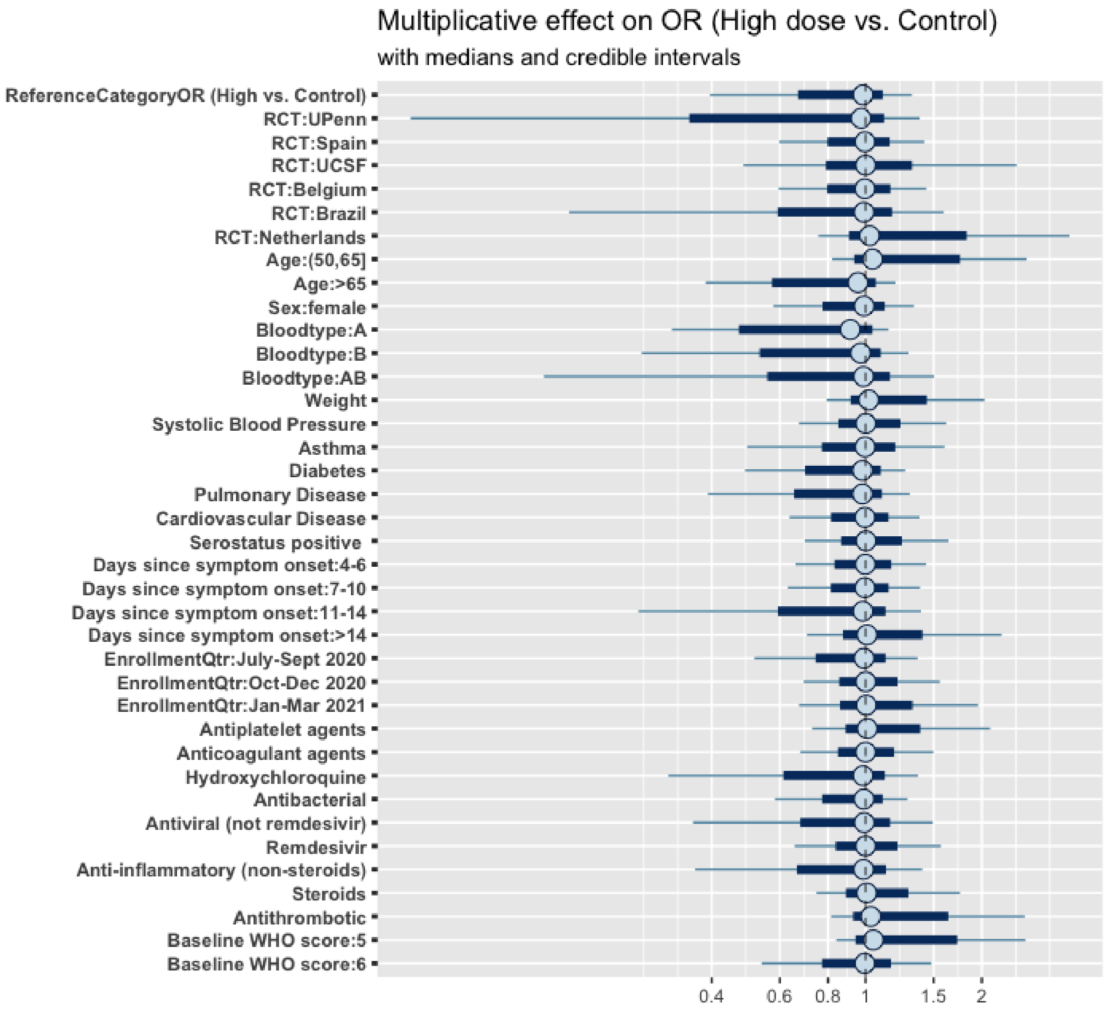

Figure S26. The estimated (exponentiated) regression coefficients (along with 80% and 95% credible intervals) of the dose group-by-baseline covariates interaction effects, each representing the corresponding baseline factor level's multiplicative impact on the efficacy OR (lower dose vs. control), compared to its reference level.

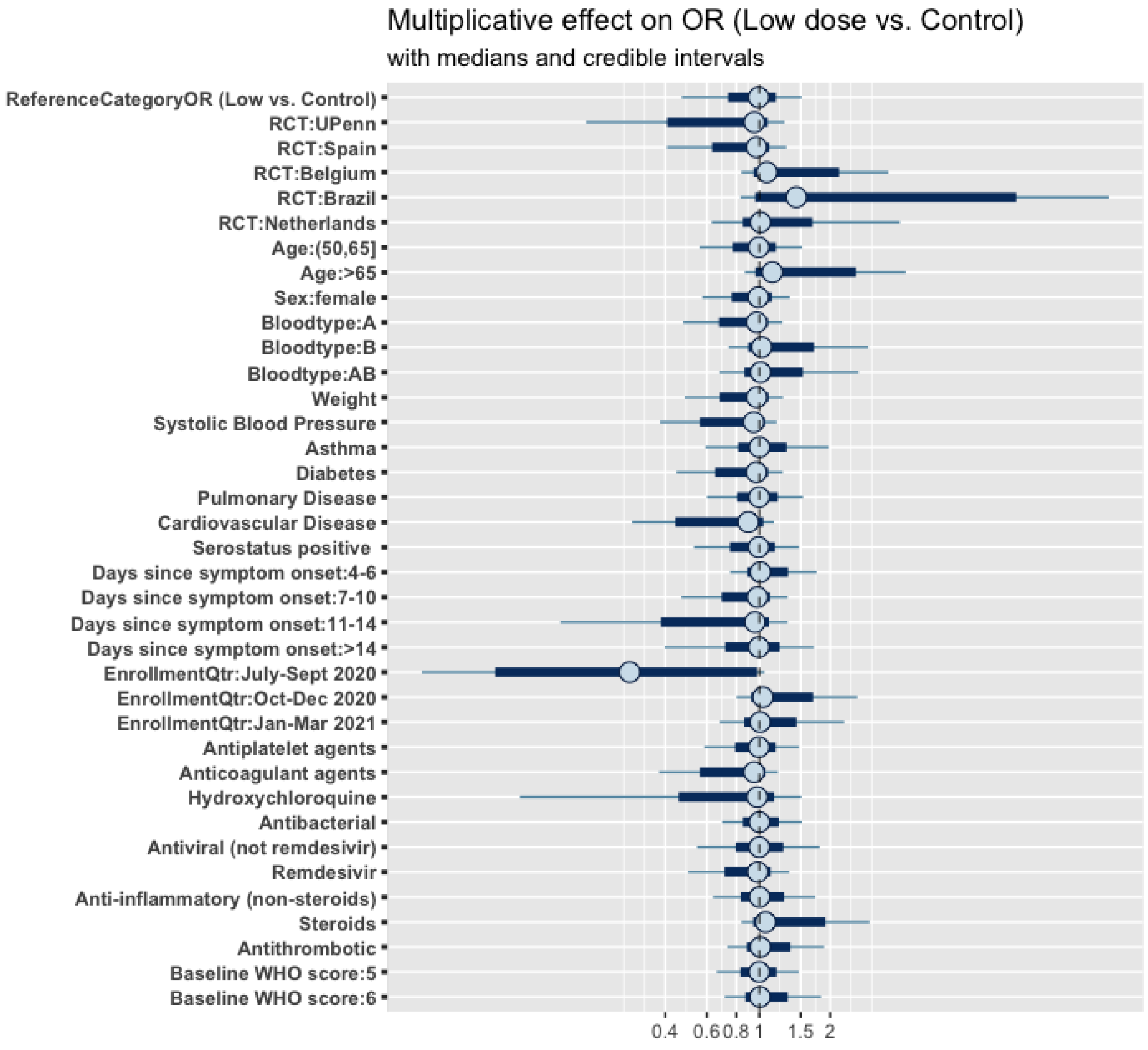

Figure S27. The estimated (exponentiated) regression coefficients (along with 80% and 95% credible intervals) of the dose group-by-baseline covariates interaction effects, each representing the corresponding baseline factor level's multiplicative impact on the efficacy OR (higher dose vs. lower dose), compared to its reference level.

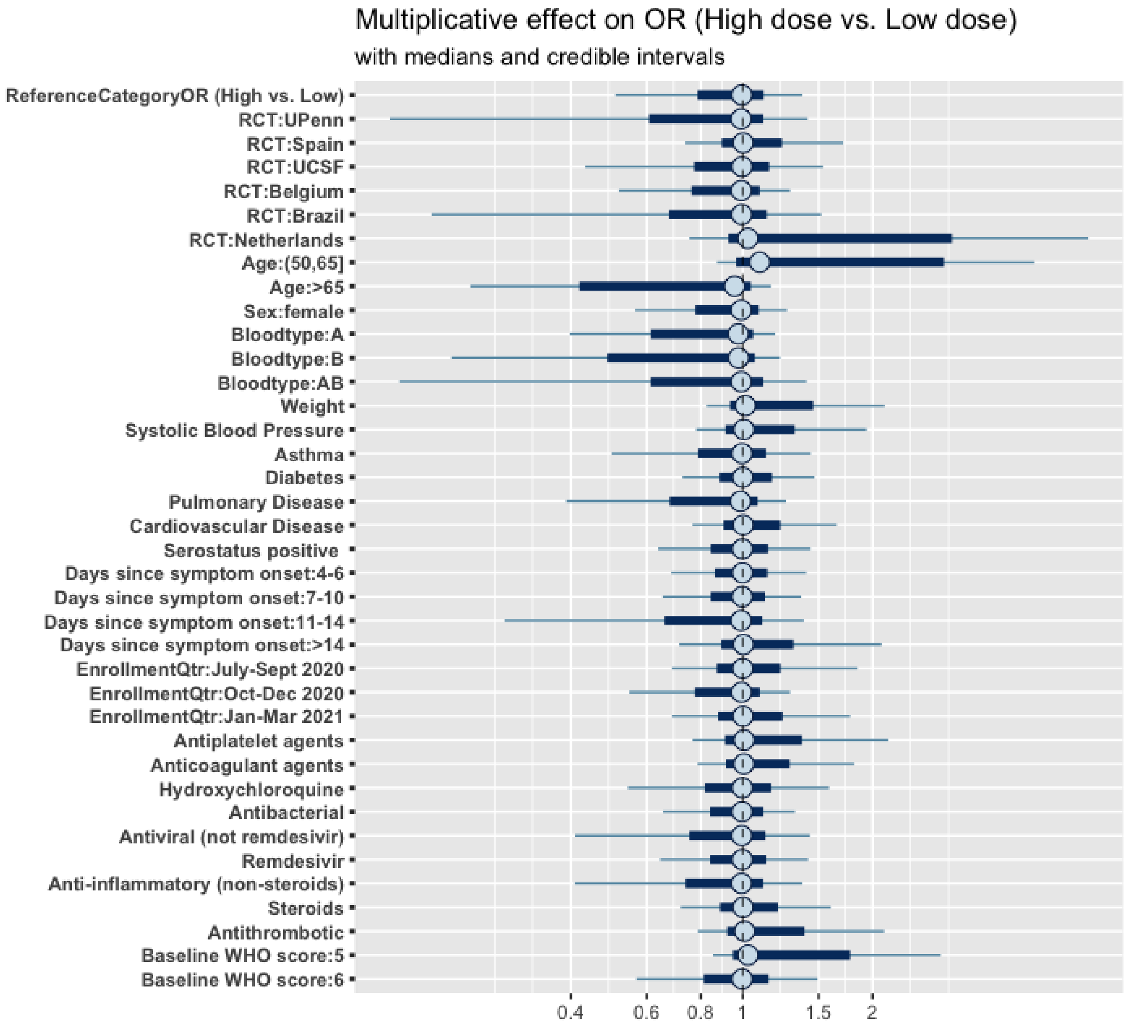

Section S4. Additional information

S4.1. Correlations of OrthoV with spike-IgG EC50 and neutralizing titer

The study utilized the semi-quantitative OrthoV assay. In a limited number of samples, one of the participating studies, CONTAIN[20], had access to quantitative measures of CCP segment, 1) CCP Segment spike-IgG half-maximal effective concentrations (EC50), measured using Einstein in-house ELISA as described previously (Yoon et al., 2020[21]; Bortz et al., 2021[22]); and 2) CCP Segment Neutralizing titers, reported as EC50 values, as described in Dieterle et al. (2020)[23]. However, CONTAIN only enrolled patients who required non-invasive oxygen supplementation at hospital admission (WHO 5 or 6), and there were no data from CONTAIN patients who did not receive oxygen supplementation at baseline (WHO 4); thus we focused on the OrthoV assay.There was a clear linear correlation between the OrthoV measure and the quantitative measures of CCP Segment spike-IgG EC50 (left panel) and Neutralizing titers (right panel), among the CONTAIN participants, as indicated in the following scatter plots.

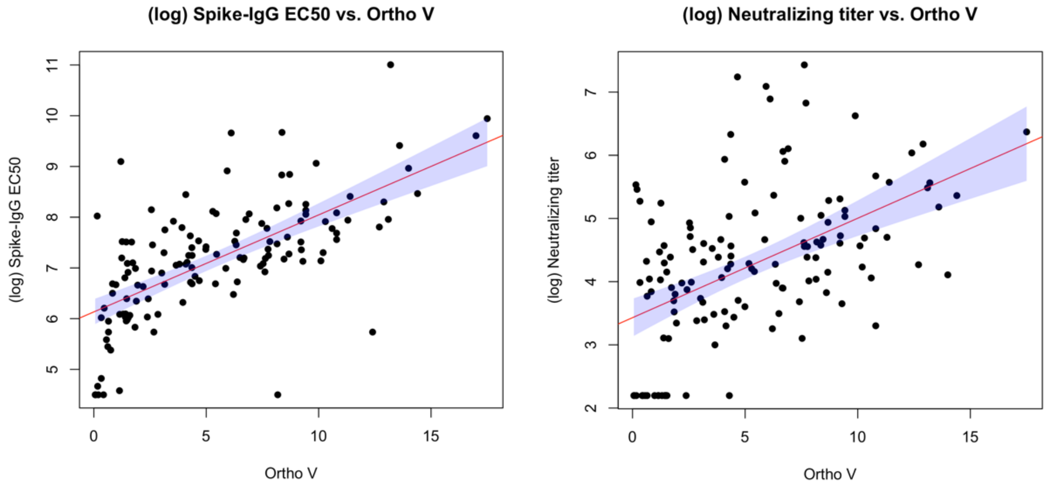

Pearson correlation coefficient between CCP Segment spike-IgG EC50 and OrthoV was 0.66 (95% CI: 0.55, 0.75) (n=135), with a Spearman correlation coefficient of 0.68. Similarly, CCP Segment Neutralizing titer and OrthoV showed a Pearson correlation coefficient of 0.51 (95% CI: [0.37, 0.62]) (n=137), and a Spearman correlation coefficient of 0.51. These results are consistent with Farnsworth et al. (2021)[24], who reported a linear relationship between OrthoV and neutralizing antibody titers. They also reported a high correlation between OrthoV and anti-spike ELISA (r = 0.90, 95% CI: [0.84, 0.93]), and a moderate correlation relative to neutralizing assays (r = 0.65, 95% CI: [0.55, 0.77]).

---

[20] Ortigoza MB, Yoon H, Goldfeld KS, …, Hendrickson JE. Efficacy and Safety of COVID-19 Convalescent Plasma in Hospitalized Patients: A Randomized Clinical Trial. JAMA Intern Med. 2022 Feb 1;182(2):115-126. PMID: 34901997

[21] Yoon HA, Bartash R, Gendlina I, …, Pirofski LA. Treatment of severe COVID-19 with convalescent plasma in Bronx, NYC. JCI Insight. 2021 Feb 22;6(4):e142270. PMID: 33476300

[22] Bortz RH 3rd, Florez C, Laudermilch E, …, Chandran K. Single-Dilution COVID-19 Antibody Test with Qualitative and Quantitative Readouts. mSphere. 2021 Apr 21;6(2):e00224-21. PMID: 33883259

[23] Dieterle ME, Haslwanter, D., Bortz III., …, Jangra, RK. A Replication-Competent Vesicular Stomatitis Virus for Studies of SARS-CoV-2 Spike-Mediated Cell Entry and Its Inhibition. Cell Host Microbe. 2020 Sep 9;28(3):486-496.e6. PMID: 32738193

[24] Farnsworth CW, Case JB, Hock K, …, Henderson JP. Assessment of serological assays for identifying high titer convalescent plasma. Transfusion. 2021 Sep;61(9):2658-2667. PMID: 34216156

S4.2. Correlations of recipients' baseline seropositive status with other variables

We correlated baseline seropositive status with: 1) baseline disease severity (WHO 4, 5, 6); 2) patients enrollment quarters; 3) the outcome of mechanical ventilation or death (WHO ≥ 7) at day 14; and 4) the outcome of death at day 28.

1) Baseline disease severity (WHO 4, 5, 6)

	Baseline WHO=4	Baseline WHO=5	Baseline WHO=6	Sum	NA's (baseline WHO)
Seronegative	56(9%)	463(77%)	85(14%)	604(100%)	0
Seropositive	34(5%)	466(68%)	187(27%)	687(100%)	0
Sum	90	929	272	1291	
NA's (serostatus)	362	572	144		1078

Pearson's Chi-squared test of association

X-squared = 38.46, df = 2, p-value = 4.451e-09 < 0.001

2) Patient enrollment quarters

	Apr-June 2020	July-Sept 2020	Oct-Dec 2020	Jan-Mar 2021	Sum	NA's (quarter)
Seronegative	62(10%)	101(17%)	294(49%)	147(24%)	604(100%)	0
Seropositive	136(29%)	96(14%)	309(45%)	146(21%)	687(100%)	0
Sum	198	197	603	293	1291	
NA's (serostatus)	443	260	306	69		1078

Pearson's Chi-squared test of association

X-squared = 22.919, df = 3, p-value = 4.199e-05 < 0.001

3) The outcome of mechanical ventilation or death (WHO ≥ 7) at day 14

	WHO < 7 at day 14	WHO ≥ 7 at day 14	Sum	NA's (WHO score at day 14)
Seronegative	523(88%)	74(12%)	597(100%)	7
Seropositive	614(90%)	69(10%)	683(100%)	4
Sum	1137	143	1280	
NA's (serostatus)	916	142		1089

The odds ratio (seropositive/seronegative) for the outcome of mechanical ventilation or death (WHO ≥ 7) at day 14 was 0.79 (95% CI: [0.56, 1.12], p-value: 0.19).

4) The outcome of death at day 28

	Alive at day 28	Death at day 28	Sum	NA's (Death at day 28)
Seronegative	493(82%)	105(18%)	598(100%)	6
Seropositive	591(87%)	92(13%)	683(100%)	4
Sum	1084	197	1281	
NA's (serostatus)	905	155		1088

The odds ratio (seropositive/seronegative) for the outcome of death at day 28 was 0.73 (95% CI: [0.54, 0.99], p-value: 0.04).

S4.3. Uniform testing of antibody titers on the OrthoV platform in COMPILE RCTs

It was of interest to evaluate the effect of titers in the transfused COVID-19 convalescent plasma (CCP) on the efficacy of the CCP treatment. The antibodies in the transfused plasma were assessed using different techniques in the different RCTs participating in COMPILE and therefore the measurements were not comparable. To be able to study effects of antibodies in the transfused CCP, it was necessary to obtain titer measurements using the same antibody test across all RCTs. To facilitate that, COMPILE investigators identified the available samples from CCP transfused to patients in their RCTs and arranged for testing of the samples on the Ortho Clinical Diagnostics VITROS® XT7600 Integrated System Anti-SARS-CoV-2 assay (OrthoV) platform.

Measurement of SARS-CoV-2 spike protein IgG levels in convalescent plasma samples

SARS-CoV-2 IgG levels were measured retrospectively on either convalescent plasma donor sera or plasma obtained at the time of donation and/or the administered convalescent plasma and associated with patient outcomes. Antibody levels were measured semi-quantitatively using the Ortho Clinical Diagnostics VITROS® XT7600 Integrated System Anti-SARS-CoV-2 assay (OrthoV) according to the manufacturer's protocol. The OrthoV platform was used to retrospectively determine SARS-CoV-2 IgG levels in donor sera from convalescent plasma units used in the Mayo Clinic Expanded Access Program study[25]. High titer convalescent plasma was authorized for emergency use for hospitalized patients with COVID-19 by the US FDA[26]. OrthoV was also the first platform authorized by the US FDA for labeling convalescent plasma units as 'high titer'[27].

Ortho Clinical Diagnostics VITROS® XT7600 Integrated System:
Anti-SARS-CoV-2 IgG assay

Sample Preparation

- **Plasma Volume**: 100uL, preferably 200uL (if needed for retest).
- **Plasma Storage**: Aliquot into either Eppendorf or cryotubes. Cryotubes with screw caps are better for shipping.
- **Plasma Processing**: Samples should be heat-inactivated at 56º C for 30 minutes and then stored at 4º C until analysis.
- **Plasma Labeling**: Identifiers (study ID, Donor Identification Number, etc.) in accordance with Study parameters can be used to label the tubes. Labeling are modeled in Ortho analysis.
- **Plasma Shipment**: Aliquots should be placed in a standard freezer box and shipped with ice packs to maintain 4º C, along with a manifest of samples.

[25] Joyner, M. J., et al. (2021). "Convalescent Plasma Antibody Levels and the Risk of Death from Covid-19." N Engl J Med 384(11): 1015-1027.
[26] US Food, Drug Administration, et al. "Recommendations for investigational covid-19 convalescent plasma." Food and Drug Administration, 2020
[27] Hinton, D. M. Letter from the US FDA https://www.fda.gov/media/141477/download
